# Acute microglia ablation induces neurodegeneration in the somatosensory system

**DOI:** 10.1038/s41467-018-05929-4

**Published:** 2018-11-01

**Authors:** Stephen J. Rubino, Lior Mayo, Isabella Wimmer, Victoria Siedler, Florian Brunner, Simon Hametner, Asaf Madi, Amanda Lanser, Thais Moreira, Dustin Donnelly, Laura Cox, Rafael Machado Rezende, Oleg Butovsky, Hans Lassmann, Howard L. Weiner

**Affiliations:** 1 Ann Romney Center for Neurological Diseases, Brigham and Women’s Hospital, Harvard Medical School, Boston, 02115 MA USA; 2 Department of Neuroimmunology, Medical University of Vienna, Spitalgasse 4, Vienna, 1090 Austria; 3 Evergrande Center for Immunologic Diseases, Brigham and Women’s Hospital, Harvard Medical School, Boston, 02115 MA USA; 4 Present Address: School of Molecular Cell Biology & Biotechnology, George S. Wise Faculty of Life Sciences, and Sagol School of Neuroscience, Tel-Aviv University, Tel-Aviv, 69978 Israel; 5 Present Address: Department of Pathology, Sackler School of Medicine, Tel Aviv University, Tel Aviv, 69978 Israel

## Abstract

Previous studies have reported that microglia depletion leads to impairment of synapse formation and these cells rapidly repopulate from CNS progenitors. However, the impact of microglia depletion and repopulation in the long-term state of the CNS environment has not been characterized. Here, we report that acute and synchronous microglia depletion and subsequent repopulation induces gray matter microgliosis, neuronal death in the somatosensory cortex and ataxia-like behavior. We find a type 1 interferon inflammatory signature in degenerating somatosensory cortex from microglia-depleted mice. Transcriptomic and mass cytometry analysis of repopulated microglia demonstrates an interferon regulatory factor 7-driven activation state. Minocycline and anti-IFNAR1 antibody treatment attenuate the CNS type 1 interferon-driven inflammation, restore microglia homeostasis and reduce ataxic behavior. Neither microglia depletion nor repopulation impact neuropathology or T-cell responses during experimental autoimmune encephalomyelitis. Together, we found that acute microglia ablation induces a type 1 interferon activation state of gray matter microglia associated with acute neurodegeneration.

## Abstract (web summary)

Previous studies have shown that depletion of microglia at early developmental stages leads to neuronal death. Here the authors use an inducible system to ablate microglia in adulthood, showing that such depletion leads to ataxia-like behavior and neuronal loss, and identifying the inflammatory components that may contribute.

## Introduction

Microglia are resident immune cells of the central nervous system (CNS) that arise from embryonic yolk sac progenitors that seed the CNS during early development^1^. Microglia are constantly surveying and interacting with neurons and other glial cells to mediate CNS homeostasis^2^. Specifically, microglia have been shown to shape synapse formation and support neurons using contact-independent mechanisms via release of growth factors and neurotrophic factor such as brain-derived neurotrophic factor (BDNF)^3^ and insulin-like growth factor 1 (IGF-1)^4,5^, and also via contact-dependent mechanisms that include CX3CR1-fractalkine^6,7^ and complement-mediated interactions^8,9^. During CNS homeostasis, adult microglia are defined morphologically by small cell bodies and numerous ramified processes, and genetically by expression of homeostatic genes including *P2ry12*, *Fcrls*, *Tmem119, Olfml3* and *Sall*^10,11^. Homeostatic microglia rapidly respond to CNS insults by migrating to and engulfing pathogens or dying cells and upon activation lose their ramified processes, proliferate and secrete cytokines and chemokines to shape the immune microenvironment in the CNS^12^.

Importantly, microglia activation is a hallmark of many neurological diseases including multiple sclerosis (MS), Alzheimer’s disease (AD), Parkinson’s disease (PD) and amyotrophic lateral sclerosis (ALS)^13–15^. However, it remains unclear whether dysfunctional microglia activation initiates and drives the pathogenesis of these diseases or whether neurodegenerative processes, such as neuronal death and protein aggregation, induce secondary microglia activation. Indeed, microglia activation pathways can have both protective or neurotoxic functions, depending on the models, timing, CNS region and techniques studied^13–15^, illustrating the complex role of these cells in mediating CNS homeostasis and the importance of defining the role of specific microglia activation states in the context of acute and chronic neurodegeneration.

In addition to being highly attuned to their environment, studies using parabiotic mice demonstrated that microglia are long-lived and exhibit a capacity for self-renewal that is unique compared to other tissue-resident macrophages such as liver Kupffer cells and intestinal macrophages^16^. Recent studies using chemical inhibitors of colony stimulating factor receptor 1 (CSF1R), a critical signal to maintain microglia survival, found that microglia self-renew from CNS progenitor cells^17^. Moreover, microglia that were ablated using CX3CR1-Cre^*ERT2*^ targeting models and fate-mapping mice confirmed that these cells form self-renewing clusters that can repopulate the CNS in 7 to 10 days^18^.

Microglia depletion using the CX3CR1-Cre^*ERT2*^ system was also reported to trigger motor learning deficits in developing pups^3^. Other studies have demonstrated that ablating microglia during embryonic or early postnatal development induces neuronal cell death in layer V cortical regions^4^. However, it remains unclear how acute microglia ablation and subsequent rapid repopulation of these cells impact on neuronal survival in adult mice and how perturbation of microglia homeostasis alters the CNS inflammatory environment in the long term.

Here, we report that diphtheria toxin (DT)-induced acute and synchronous microglia depletion in adult mice using the CX3CR1-CreER system triggered gray matter gliosis associated with progressive ataxia-like neurological behavior. Notably, microglia-depleted mice exhibited severe injury and loss of neuronal cells in the somatosensory system including the dorsal horn of the spinal cord, the thalamic relay nuclei and the layer IV of the somatosensory cortex. Transcriptomic analysis demonstrated that neurodegeneration was accompanied by activation of the type 1 interferon response. Repopulated microglia isolated from these mice exhibited an interferon regulatory factor 7 (IRF7)-driven activation state and we found that minocycline treatment or blocking type 1 interferon signaling rescued mice from ataxic behavior. Finally, acute microglia depletion and repopulation affect mortality and clinical signs in experimental autoimmune encephalomyelitis (EAE), but does not impact on lesion pathology or the CNS T-cell response and did not alter the neurodegenerative phenotype in the somatosensory system. Taken together, our results demonstrate that severe and synchronous microglia perturbation by DT-mediated ablation induces gray matter neuronal death in adult mice, which is driven by an in vivo type 1 interferon signature.

## Results

### Acute microglia ablation triggers ataxia-like behavior

**Fig. 1:**
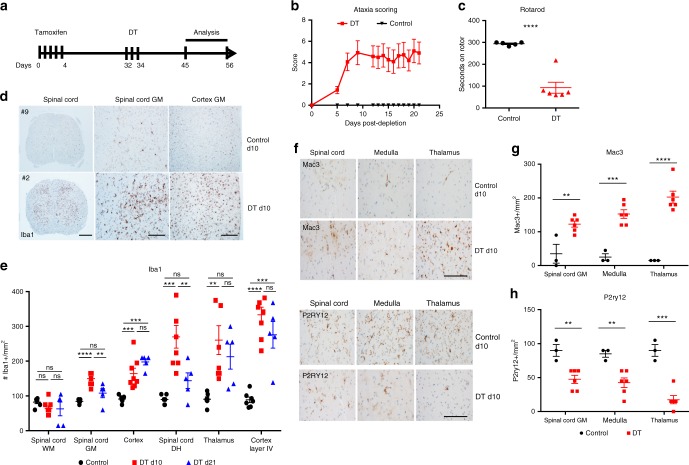
Microglia depletion triggers ataxia and gray matter microgliosis. **a** Schematic outline of TAM and DT treatment strategy to study impact of microglia depletion on neuroinflammation. Microglia-depleted mice (red) exhibited **b** severe ataxia behavior scores compared to littermate control mice (black). **c** Dot plot depicts the seconds on rotarod of mice at d10 post depletion (red) and littermate controls (black). **d** Representative images of Iba1 immunohistochemical staining in spinal cord and somatosensory cortex at d1 post depletion and control animals. Scale bar for spinal cord left column = 200 μm and scale bar for middle and right column = 100 μm. **e** Dot plots depict the average number of Iba1^+^ cells per mm^2^ quantified in control (black), d10 post microglia depletion (red) and d21 post microglia depletion (blue) in spinal cord gray matter (GM), spinal cord white matter (WM), the frontal cortex and the somatosensory system, including the dorsal horn of the spinal cord, the thalamus and the layer IV of the somatosensory cortex. Representative immunohistochemistry of the activation marker Mac3 (**f**) and the homeostatic microglia marker P2ry12 (**h**) in the gray matter of the spinal cord, medulla and thalamus from control and ataxic mice at day 10 post depletion, scale bar = 100 μm. Dot plots depict the average number of Mac3^+^ (**g**) and P2ry12^+^ (**i**) cells per mm^2^ quantified in control (black) and d10 post microglia depletion (red). Representative experiment of three independent experiments. Error bars depict SEM and *n* = 3–7 mice per group. **e** Analyzed by one-way ANOVA with Tukey’ post-hoc test for multiple comparisons and **g**, **i** analyzed with Student’s unpaired *t*-test at 95% confidence interval and ****p < 0.0001, ***p < 0.001, **p < 0.01, ns not significant

To deplete microglia, we crossed tamoxifen (TAM)-inducible CX3CR1-Cre^*ERT2*^ mice with flox-STOP-diphtheria toxin receptor mice (iDTR) (Supplementary Fig. 1a). TAM injection in CX3CR1-Cre^*ERT2*+/–^/iDTR^+/–^ mice induced the transient expression of a Cre-recombinase under the CX3CR1 promoter that excised the flox-flanked STOP sequences upstream of the DTR sequence leading to expression of the toxin receptor on the surface of CX3CR1-GFP expressing cells (Supplementary Fig. 1b). Microglia express very high levels of CX3CR1 and have been shown to be specifically targeted using this system by administrating TAM, waiting a minimum of 28 days for peripheral monocytes to be replaced de novo by non-targeted bone marrow-derived progenitors and then injecting DT (Fig. 1a). Using this strategy, we confirmed by immunohistochemical staining of Iba1 (myeloid marker), P2RY12^10,19^ and Mac3 (microglia/macrophage activation marker) in the cortex (Supplementary Fig. 1b) and by flow cytometry that over 95% of microglia (defined as CX3CR1-GFP^+^CD45^int^CD11b^+^Ly6C^–^) were specifically depleted in the brain at day 1 (d1) post DT injection, whereas cells in the spleen were not ablated (Supplementary Fig. 1c–d). As additional controls, we found similar levels of CX3CR1-GFP^+^CD45^+^CD11b^+^Ly6C^–^ cells at d1 post DT in the mesenteric lymph nodes, cervical lymph nodes, liver, bone marrow and colon lamina propria (Supplementary Fig. 1e). Only in the small intestine lamina propria was there a reduction in the percentage CX3CR1-GFP^+^CD45^+^CD11b^+^Ly6C^–^ cells at d1 post DT (Supplementary Fig. 1e) and this could be caused by the slower turnover of intestinal myeloid cells that has been previously reported^20^.

Microglia-depleted mice progressively developed severe motor coordination dysfunction similar to spinocerebellar ataxia, including kyphosis, loss of balance and hind limb clasping (Fig. 1b). Ataxic behavior typically began at day 7 and peaked at days 10–14 post depletion. Accordingly, microglia-depleted mice at d10 compared to littermate controls exhibited severe loss of motor function as measured by rotarod testing (Fig. 1c). We observed striking microgliosis in the gray matter of the spinal cord and brain (Fig. 1d, e) by immunohistochemical staining of the microglia marker Iba1. This activation persisted until d21 post depletion. Interestingly, we did not observe increased Iba1 reactivity at d10 or d21 post depletion in the white matter (Fig. 1d, e), suggesting regional differences in restoration of microglial homeostasis after perturbation. Microglial activation was most pronounced in the somatosensory system, including the laminae 1 and 2 of the dorsal horn of the spinal cord, the thalamic relay nuclei and the layer IV of the somatosensory cortex (Fig. 1e, Supplementary Fig. 1f). We found increased expression of the Mac3 (Fig. 1f, g) and lower expression of P2RY12 (Fig. 1h, i) in the gray matter of the spinal cord, medulla and thalamus. Moreover, at d3 post depletion we observed proliferating Iba1^+^ cell clusters confirming the self-renewal capacity of these cells reported in previous studies^17,18,21^ (Supplementary Fig. 2a–b). Accordingly, only microglia from d3 post-depleted mice were proliferating as determined by PCNA^+^ immunohistochemistry and these proliferating clusters were confirmed to be microglia as they were all TMEM119+, a marker microglia-specific marker^10,22^ (Supplementary Fig. 2c, d). We also discovered a modest recruitment of Ly6C^hi^CD11b^+^CD45^hi^ monocytes that peaked at d1 post depletion (Supplementary Fig. 3a–c). Importantly, Ly6C^hi^CD11b^+^CD45^hi^ did not express the microglia markers TMEM119 or CD39, indicating that these were indeed recruited monocytes and Ly6C expression was not upregulated on repopulated microglia (Supplementary Fig. 3d, e). Together, our results demonstrate that the proliferating clusters of remaining cells are microglia; however, a small number of recruited monocytes could be contributing to the pool of repopulated cells.

**Fig. 2:**
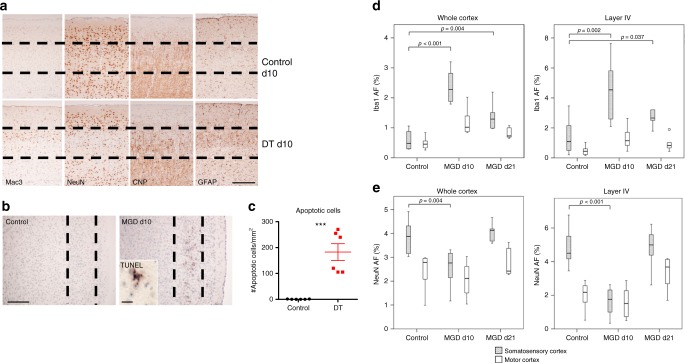
Degeneration of layer IV somatosensory neurons after microglia ablation. **a** Representative immunohistochemistry staining for Mac3, NeuN, CNP and GFAP in the somatosensory cortex in control and d10 post depletion mice. Microglia-depleted animals show increased numbers of Mac3- and GFAP-positive cells, but decreased numbers of NeuN-reactive cells. Dashed lines outline layer IV and scale bar = 100 μm. **b** TUNEL staining in the somatosensory cortex in control and d10 post depletion animals. Dashed line outlines layer IV, scale bar = 100 μm and 10 μm for cropped TUNEL image. **c** Dot plot depicts quantification of number of apoptotic cells per mm^2^ in layer IV somatosensory cortex of d10 post depletion mice (red) and littermate controls (black). Error bars depict SEM and *n* = 5–7 mice group. Analyzed with Student’s unpaired *t*-test at 95% confidence interval and ***p < 0.001. **d**, **e** Densitometric quantification of Iba1 (**d**) and NeuN (**e**) immunoreactivity depicted from whole cortex and layer IV in control, d10 and d21 post depletion animals (gray bars = somatosensory cortex, white bars = motor cortex, MGD = microglia depleted). Box plots depict medians, interquartile ranges (IQR, boxes) and values within 1.5× IQR away from the respective quartile (whiskers). Values that lie outside 1.5× IQR are depicted as circles. Analyzed with Kruskall–Wallis test followed by Mann–Whitney test and corrected for multiple comparisons with Bonferroni Holm test. Staining for the microglia marker Iba1 shows a profound increase of microglia in the somatosensory cortex and in particular in layer IV in animals at day 10 after microglia depletion in comparison to that in control animals or even in the motor cortex of microglia-depleted animals. Neuronal density in the normal cortex and in particular in layer IV is more pronounced in the somatosensory (granular) compared to the motor (agranular) cortex. Significant neuronal loss is only seen in the granular cortex and in particular in layer IV at day 10. The density of neurons in the somatosensory cortex shows comparable values in comparison to controls at day 21 due to profound shrinkage and atrophy of layer IV at this time point

We observed a significant loss of NeuN^+^ neurons in the somatosensory cortex at d10 post microglia depletion (Fig. 2a). The TUNEL^+^ (terminal deoxynucleotidyl transferase-dUTP nick end labeling-positive) neurons were localized to layer IV of the somatosensory cortex in the ataxic mice at d10 post depletion (Fig. 2b, c), indicating that microglia ablation and subsequent repopulation triggered neuronal apoptosis at this time point. Increased Mac3 staining was observed specifically in the layer IV compared to other layers of the cortex suggesting layer-specific microgliosis (Fig. 2a). Moreover, we found increased GFAP (glial fibrillary acidic protein) but not CNP (2′,3′-cyclic-nucleotide 3′-phosphodiesterase) staining in the somatosensory cortex of d10 post-depleted mice (Supplementary Fig. 4), indicating that microglia ablation triggers astrogliosis but does not impact oligodendrocytes in this region. Densitometric analysis of either the whole cortex or only layer IV demonstrated increase in Iba1 immunoreactivity in the somatosensory and—less pronouncedly—in the motor cortex at d10 post depletion. Increased Iba1 immunoreactivity persisted until d21 (Fig. 2d). Conversely, densitometry of NeuN staining revealed a profound loss of reactivity at d10 but not d21, which was due to atrophy of the entire layer IV by d21 post depletion (Fig. 2e). Importantly, at d1 and d3 post DT injection, there was no observable loss of NeuN staining in the somatosensory cortex (Supplementary Fig. 2d), indicating that the neurodegeneration results from a secondary insult that occurs after microglia depletion and not from direct neurotoxicity by diphtheria toxin. Similar changes, but less severe, were also present in the dorsal horn of the spinal cord and in the somatosensory relay nuclei of the thalamus (data not shown).

Together, our results suggest that microglia rapidly repopulate the CNS in adult mice after acute and synchronous DT-mediated depletion, and this is associated with a loss of neuronal homeostasis in the somatosensory system.

### Type 1 interferon signature of acute neurodegeneration

**Fig. 3:**
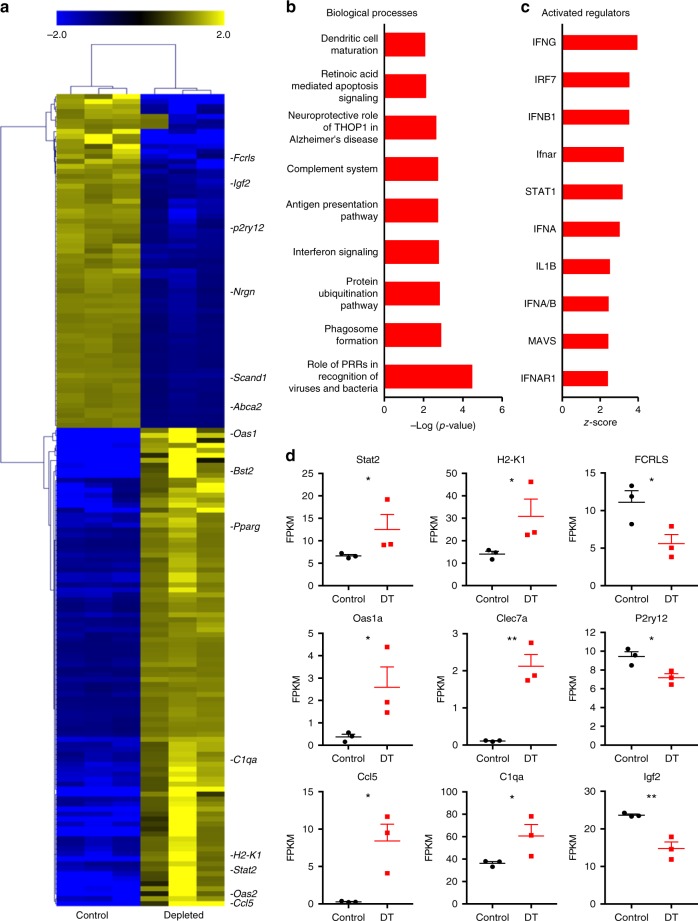
Type 1 interferon inflammatory signature associated with acute neurodegeneration. **a** Heatmap depicts hierarchical clustering of upregulated (yellow) and downregulated (blue) genes in cortical tissue from d10 microglia-depleted mice identified by DeSEQ2 analysis of TMM normalized RNA-Seq values. **b**, **c** Bar graphs depict Ingenuity pathway analysis of the 10 most significant biological processes and predicted upstream regulators of the DE genes in the dataset. **d** Dot plots demonstrate the FPKM (fragments per kilobase million) values in cortical tissue from control (black) and depletion (red). Cortical tissue from ataxic mice demonstrated upregulation of type 1 interferon pathway genes and genes associated with microglia activation, and lower expression of genes associated with a neuronal and microglia homeostasis. Error bars depict SEM and *n* = 3 mice per group. Analyzed with Student’s unpaired *t-*test at 95% confidence interval and *p < 0.05, **p < 0.01

To gain further insights into the pathways driving neurodegeneration and acute microgliosis specifically in our model, we isolated RNA from frontal cortical tissues of d10 depleted and non-depleted littermate control mice and performed RNA sequencing (RNA-seq) analysis. We identified 163 differentially expressed (DE) genes using the DeSEQ2 algorithm (Fig. 3a). Of these, 66 genes were significantly downregulated and 97 were upregulated (Fig. 3a). Using Ingenuity pathway analysis (IPA), we found that the DE genes were strongly associated with inflammatory processes, including pattern recognition signaling, interferon signaling, complement system and antigen presentation pathways (Fig. 3b). Strikingly, 8 of the 10 top activated regulators that were predicted using IPA were type 1 interferon signaling genes (Fig. 3c) including *Irf7, Ifnb1, Ifnar* and *Stat1* and which were strongly predicted to be induced by the anti-viral response (Supplementary Fig. 5). Moreover, many of the genes that were upregulated in our dataset are involved in the type 1 interferon signaling network, including *Stat2, Oas1a* and *Ccl5* (Fig. 3d, Supplementary Fig. 5a). Conversely, most of the downregulated genes were linked to loss of neuronal homeostasis (Supplementary Fig. 5b), including downregulation of homeostatic microglia molecules *Fcrls* and *P2ry12* as well as neuronal homeostasis mediators such as *Igf1* and *Abca2* and upregulation of *Pparγ* expression (Supplementary Fig. 5b).

To further investigate the gene signature we observed in the RNA-seq analysis, we performed microarray analysis on laser-microdissected layer IV tissue from the somatosensory cortex from microglia-depleted and littermate control mice. To serve as an intrinsic control, we analyzed layer IV of the motor cortex from the same animals (Supplementary Fig. 6a–b). We found that the transcriptomic signature of these cortical layer-specific analyses also demonstrated upregulation of innate inflammatory pathways including type 1 interferon signaling (Supplementary Fig. 6c–d). Similar to the RNA-seq data, the majority of the upstream regulators predicted by Ingenuity analysis of the differentially expressed genes in our microarray dataset targeted the type 1 interferon signaling pathway, including *Stat1, Irf7, Ifnar, Ifna1, Ifna2* and *Ifnb1* (Supplementary Fig. 6e).

To investigate the type 1 interferon signature at the protein level, we stained for the interferon-dependent protein OAS1A in microglia-depleted and control animals at d10 post depletion. OAS1A expression was significantly upregulated in microglia and neurons of layer IV of the somatosensory cortex of microglia-depleted animals compared to control animals as well as compared to layer IV of the motor cortex of microglia-depleted and control mice (Supplementary Fig. 7a–b). These results demonstrate a type 1 interferon response specific to regions associated with neuronal death. We next stained for nucleic acids using the 9D5 antibody; nucleic acids are products of dying cells and induce type 1 interferon responses. We observed increased 9D5+ neurons in areas of neurodegeneration, including layer IV of the somatosensory and motor cortex in microglia-depleted mice, but not in control animals (Supplementary Fig. 7c–d).

Taken together, these results demonstrate that a type 1 interferon signaling activation after acute microglia ablation is associated with somatosensory neurodegeneration.

### IRF7-driven activation state of repopulated microglia

**Fig. 4:**
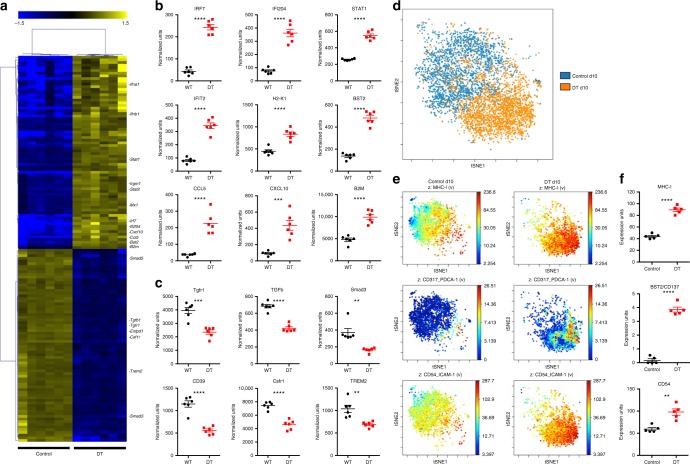
Loss of homeostatic microglia after depletion. CD11b^+^CD45^int^CX3CR1-GFP^+^Ly6C^–^ microglia were sorted from d10 control and d10 post microglia depletion mice and their transcriptome was analyzed using the Nanostring mouse immunology kit. **a** Heatmap depicts hierarchical clustering of significantly upregulated (yellow) and downregulated (blue) genes in microglia from six control and six d10 microglia-depleted mice. **b**, **c** Dot plots demonstrate the relative expression levels in wild-type control (black bars) and d10 post depletion microglia (red bars). **b** Microglia from ataxic mice demonstrated upregulation of type 1 interferon pathway genes: *IRF7, Ifi204, Stat2, Ifit2, H2-K1, B2M, BST2, CCL5, CXCL10*. **c** Microglia from ataxic mice demonstrated lower expression of genes associated with a homeostatic microglia signature *TGFb*, *Tgfr1*, *Smad3, CD39 (Entpd1), Csfr1* and *Trem2*. To assess the activation state of repopulated microglia, control and d10 post depletion CX3CR1-GFP^+^CD45^int^CD11b^+^Ly6C^–^ microglia were analyzed by CyTOF. **d** ViSNE analysis of control and repopulated microglia demonstrated that repopulated microglia cluster differently than controls, indicating a unique activation state of these cells. **e** Expression of MHCI, CD137/BST2 and CD54 in control and repopulated microglia clusters. **f** Dot plots depict quantification of CyTOF expression units for MHCI, CD137/BST2 and CD54 in control (black) and d10 post depletion microglia (red). Representative experiment of two independent experiments. Error bars depict SEM and *n* = 5–7 mice per group. Analyzed with Student’s unpaired *t*-test at 95% confidence interval and ****p < 0.0001, ***p < 0.001, **p < 0.01

In order to investigate the activation state of the repopulated microglia, we sorted CX3CR1-GFP^+^CD45^int^CD11b^+^Ly6C^–^ microglia from d10 post depletion and littermate control mice and measured their activation gene profile using a Nanostring array of 560 immunology-associated genes. Hierarchical clustering analysis of 149 differentially expressed genes revealed a clear distinction between repopulated microglia at d10 compared to littermate controls (Fig. 4a). The 77 upregulated genes were associated with microglia/macrophage activation including RF7, IRF7-dependent genes (*Ifi204*, *Stat2* and *Ifit2*), surface activation markers major histocompatibility complex (MHC) class I (*H2-k1*) and *Bst2*, and type 1 interferon chemokines such as *Ccl5* and *Cxcl10* (Fig. 4b). Many of the downregulated genes were homeostatic microglia genes including the transforming growth factor-β (TGF-β) signaling pathway (*Tgfr1*, *Tgf-β, Smad3*) and surface markers such as *Entpd1* (CD39), *Csfr1* and *Trem2* (Fig. 4c). Importantly, we have previously shown that TGF-β signaling is critical to maintain microglia homeostasis and deletion of CNS TGF-β expression also leads to severe neurological pathology^10^.

Mass cytometry (cytometry by time of flight (CyTOF)) has recently been shown to be an effective method to characterize myeloid cell subsets^23^. We used a CyTOF panel of 33 monocyte/macrophage markers (Supplementary Table 1) to characterize the high-dimensional surface marker expression of microglia at d10 post depletion. Using unbiased ViSNE clustering analysis, we observed a marked difference in clustering of the gated microglia population (Supplementary Fig. 8) between microglia-depleted/repopulated and control mice at d10 post deletion (Fig. 4d), indicating that the repopulated microglia have an altered phenotype compared to control microglia. Specifically, numerous activation surface markers, including MHCI, BST2 and CD54, were significantly upregulated on repopulated microglia (Fig. 4e, f). These results are consistent with the transcriptomic changes we observed in repopulated microglia, and together define a type 1 interferon activation signature of microglia associated with neurodegeneration.

### Blunting inflammation reduces microglia ablation-triggered ataxia

**Fig. 5:**
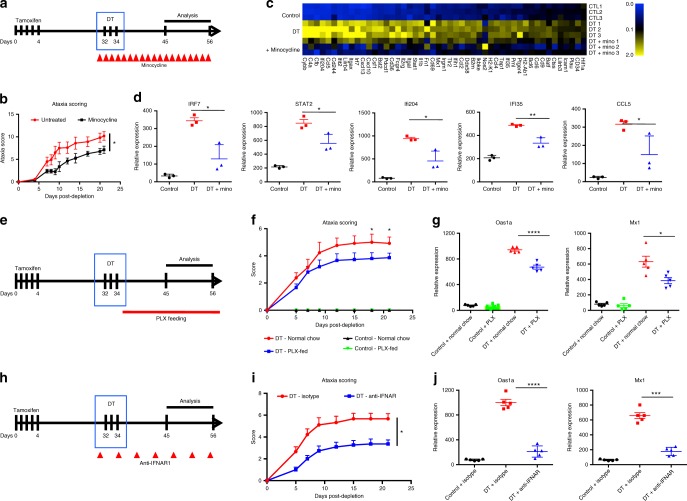
Blunting type 1 interferon-mediated neuroinflammation reverses effects of microglia ablation. **a** Schematic of minocycline treatment to blunt neuroinflammation in microglia-depleted mice. **b** Ataxia behavior scoring of microglia-depleted mice (red) and minocycline-treated mice (black). **c** Heatmap depicts upregulated (yellow) and downregulated (blue) genes in microglia from control, d10 microglia-depleted and d10 microglia-depleted mice treated with minocycline analyzed with Nanostring immunology chip. **d** Dot plots demonstrate the relative expression levels in wild-type control (black), d10 post depletion microglia (red) and d10 microglia-depleted mice treated with minocycline (blue). **e** Schematic of PLX5562 feeding to prevent microglia repopulation in DT-ablated mice. **f** Ataxia clinical scoring of DT mice treated with isotype control (red) or anti-IFNAR1 antibody (blue). **g** Dot plots demonstrate the relative expression levels of the *Oas1a* and *Mx1* in cortical tissue from non-depleted control mice fed control chow (black), control mice fed PLX5562 chow (green), d10 post depletion fed normal chow (red) and d10 post depletion fed PLX chow (blue). **h** Schematic of anti-IFNAR1 treatment to blunt type 1 interferon signaling in DT-ablated mice. **i** Ataxia clinical scoring of DT mice treated with isotype control (red) or anti-IFNAR1 treated mice (blue). **j** Dot plots demonstrate the relative expression levels of the *Oas1a* and *Mx1* in cortical tissue from control (black), d10+ isotype control post depletion (red) and d10 post depletion+anti-IFNAR1 (blue). Representative experiment of two independent experiments. Error bars represent SEM, *n* = 3–5 mice per experiment and dot plots analyzed with unpaired *t*-test at 95% confidence interval, ****p < 0.0001, ***p < 0.001, **p < 0.01, *p < 0.05

To further investigate the link between the CNS inflammatory response that is induced after microglia depletion and the subsequent neurodegeneration, we treated depleted and control mice with minocycline, a compound that has been extensively shown to limit neuroinflammation^24–26^ (Fig. 5a). Minocycline treatment significantly attenuated ataxia behavior after microglia depletion (Fig. 5b). Gray matter microglia activation as measured by CD68 staining was also attenuated in minocycline-treated mice (Supplementary Fig. 9a–b). However, minocycline treatment did not impact the number of Iba1+ cells that repopulated in gray matter of spinal cord, cortex, layer IV or thalamus (Supplementary Fig. 9c) and did not appreciably reduce neuronal apoptosis in somatosensory cortex at d10 post depletion (Supplementary Fig. 9d). Moreover, minocycline treatment suppressed *Irf7* and the IRF7 responsive genes *Ifi204, Stat2, Ifi35* and Ccl5 (Fig. 5c, d) and the surface activation markers *H2-k1* (MHC class I), *Bst2* and *B2m* (Supplementary Fig. 9e) in microglia sorted from d10 post depletion compared to littermate controls. These findings suggest that the CNS inflammation induced after microglia depletion is necessary for ataxia disease progression.

To determine whether the neuroinflammatory and ataxia phenotypes we observed following microglia depletion were associated with microglial repopulation, we treated DT-depleted mice with the CSFR1 inhibitor PLX5562 (Plexxikon) (Fig. 5e). PLX5562 depletes microglia^21^ and thus would prevent the microglia from repopulating. We found that either control or DT-depleted mice fed the PLX5562 containing chow had reduced numbers of CX3CR1-gfp+ microglia as measured by flow cytometry of whole CNS (Supplementary Fig. 10a). Furthermore, PLX5562 treatment reduced ataxia at d18 and d21 post ablation (Fig. 5f) and reduced type 1 interferon-mediated neuroinflammation at d10 post ablation, as measured by expression of *Oas1a* and *mx1* at d10 post DT (Fig. 5g). Control mice fed PLX5562 diet did not demonstrate signs of ataxia or exhibit upregulation of type 1 interferon responsive genes (Fig. 5f, g), indicating that the phenotype is specific to the acute and synchronous nature of the DT-mediated depletion. While PLX5562 was effective in preventing repopulation of the CNS as measured by flow cytometry, we found more Iba1+ cells by immunohistochemistry (IHC) staining in the somatosensory cortex of DT-depleted PLX5562 mice compared to DT-depleted mice fed a control diet (Supplementary Fig. 10b, d), indicating that the repopulating microglia associated with the layer IV neurodegeneration are partially resistant to CSFR1 inhibition, which may be related to their reduced expression of the receptor (Fig. 4c). We also found that PLX5562 treatment reduced neuronal cell death in the somatosensory cortex at d10 post DT (Supplementary Fig. 10c, d), demonstrating that preventing repopulation of activated microglia attenuates the rate of neuronal loss. Taken together, these results indicate that preventing repopulation of activated microglia reduces the pathology associated with acute DT-mediated microglia depletion.

In order to definitively establish that a type 1 interferon response was crucial for the effects we observed following microglial depletion, we blocked the type 1 interferon pathway using anti-IFNAR antibodies. We found that blocking the type 1 interferon pathway (Fig. 5h) reversed both the ataxia (Fig. 5i) and decreased the expression of the type 1 interferon genes *Oas1a* and *Mx1* in cortical tissue (Fig. 5j). These results indicate that the acute and synchronous ablation of microglia triggers a pathogenic type 1 inteferon-mediated neuroinflammatory response.

### Acute microglia depletion and repopulation in EAE

**Fig. 6:**
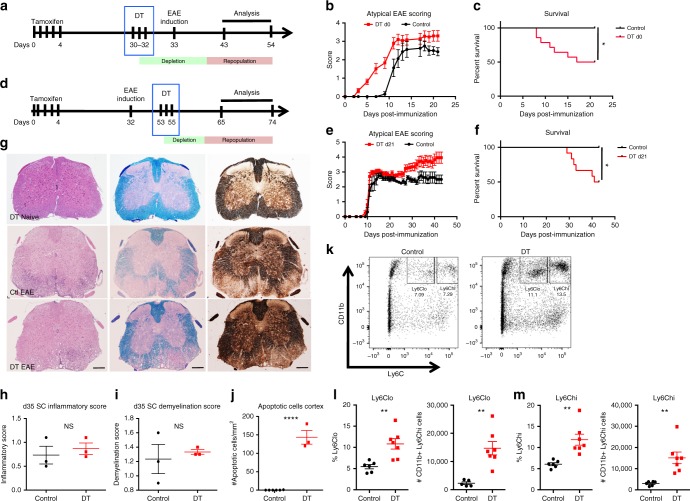
Impact of microglia ablation on experimental autoimmune encephelomyelitis. **a** Schematic of microglia depletion before MOG/CFA-induced EAE. In this experimental paradigm, microglia are depleted preceding immunization. **b** Atypical EAE disease course and **c** mortality of microglia-depleted mice (red) compared to littermate control mice (black) (*n* = 9–13 mice per group). **d** Schematic of microglia depletion at d21 post induction of MOG/CFA-induced EAE. In this experimental paradigm, microglia are depleted during the initial phase and inflammatory peak in EAE. **e** Atypical EAE disease course and **f** mortality of microglia-depleted mice (red) compared to littermate control mice (black) (*n* = 9–15 mice per group). **g** Representative IHC of H&E, Luxol Fast Blue and Silver staining in spinal cord (SC) of naive d10 microglia-depleted mice, d35 EAE from control and mice with microglia ablated at d21 post-EAE induction showing similar EAE-related pathology of inflammation, demyelination and axonal loss between control and microglia-depleted animals. Scale bar = 200 μm. **h**, **i** Quantification of inflammatory score (**h**) and demyelination score (**i**) of SCs at d35 post EAE induction. **j** Dot plot of number of apoptotic cells per mm^2^ in somatosensory cortex at d35 EAE. Representative experiment of two independent experiments. **k** Dot plot of CD11b^+^Ly6^lo^ and CD11b^+^Ly6C^hi^ infiltrating monocytes in the brain at d35 EAE. **l**, **m** Quantification of percentage and cell number of CD11b^+^Ly6C^lo^ (**l**) and CD11b^+^Ly6C^hi^ (**m**) from control and microglia-depleted EAE mice. Error bars depict SEM and *n* = 5–7 mice per group; ****p < 0.0001, ***p < 0.001, **p < 0.01, *p < 0.05 and NS not significant

During EAE, microglia activation is known to precede the onset of lymphocytic inflammation, demyelination, neurodegeneration and clinical signs of disease. We therefore investigated whether the major perturbation of microglia homeostasis would impact acute T cell-driven CNS inflammation. To address this question, we first depleted microglia in CX3CR1-Cre^ERT2+/–^iDTR^+/–^ mice prior to myelin oligodendrocyte glycoprotein (MOG) immunization for EAE. Although we observed worse ataxia clinical signs and increased mortality when microglia were depleted at this time point (Fig. 6a–c), there was no effect on spinal cord pathology as measured by demyelination, CD3, Mac3 and Iba1 staining (Supplementary Fig. 11a, b). Furthermore, EAE-induced T cell-mediated inflammation did not affect the extent and patterns of neurodegeneration in the somatosensory system as we observed increased numbers of apoptotic neurons in DT-depleted mice similar to what we found in the naive state (Supplementary Fig. 11c). Microglia-depleted animals demonstrated signs of ataxia as early as day 3 post induction, which precedes the onset of classic paralytic signs observed in EAE (Fig. 6b). Microglia depletion prior to EAE induction had no effect on peripheral T-cell responses (Suppl. Fig. 12b–d).

We next depleted microglia in CX3CR1-Cre^ERT2+/–^iDTR^+/–^ mice at the peak of disease (d21) and found that ablation increased mortality and induced disease score (Fig. 6d–f). In addition, to measures of paralysis, we also scored the mice for balance and ataxia, as these were a prominent feature of clinical disease, and are similar to those seen after microglia depletion alone. Surprisingly, microglia depletion at peak EAE had no effect on spinal cord pathology as measured by inflammation, demyelination and axonal injury (Fig. 6g–i). On the other hand, we observed neuronal cell death in the somatosensory system of microglia-depleted animals (Fig. 6j), which mimicked the pathology observed in non-immunized microglia-depleted mice. Depletion at peak EAE did not impact the CNS T-cell response, where we observed similar levels of T helper type 1 and 17 (Th1 and Th17) cells in the spinal cords of both depleted and control mice (Supplementary Fig. 12d, e). Microglia depletion during peak EAE resulted in the loss of CX3CR1-GFP/CD11b^+^CD45^int^ microglia cells (Supplementary Fig. 12f, g) and induced robust recruitment of CD45^hi^CD11b^+^Ly6C^Hi^ and CD45^hi^CD11b^+^Ly6C^lo^ inflammatory monocytes to CNS (Fig. 6l, m). These results demonstrate that microglia depletion in a CD4^+^ T cell-mediated inflammatory CNS environment does not impact CNS T-cell responses directly, but does result in an increased influx of Ly6C^hi^CD11b^+^ inflammatory monocytes and delayed repopulation of CX3CR1-GFP/CD11b^+^CD45^int^ microglia compared to naive mice, which is line with previous work demonstrating differing dynamics of CNS myeloid cell recruitment and proliferation under defined host inflammatory conditions^27–29^.

Taken together, microglia depletion in the context of EAE does not affect the development and severity of white matter T-cell inflammation or change the pattern of neurodegeneration in the somatosensory system.

## Discussion

We have found that acute and synchronous microglia depletion followed by repopulation with a dysregulated type 1 interferon activation phenotype contributes to neurological dysfunction and acute neurodegeneration. Specifically, severely perturbing microglia homeostasis by DT-mediated ablation in adult mice triggers a type 1 interferon-defined inflammatory cascade that results in neurodegeneration. These results expand upon previous work that showed neurodegeneration in layer V cortex as a consequence of microglia perturbations during embryonic and early postnatal (d1–3 after birth) development^4,30^. However, the inflammatory environment in these studies was not examined.

Moreover, we identified an IRF7-driven activation signature in repopulating microglia isolated from ataxic mice. Importantly, this activation signature did not follow the classic M1/M2 myeloid cell activation paradigm. Other studies have also reported unique microglia activation states in neurodegenerative diseases^31^. Recently, higher expression levels of IRF7 and type 1 interferon pathways were found to be associated with the aging brain, cognitive decline and neurodegeneration^32^. Indeed, IRF7 and downstream genes such as *Ifit2* and *Bst2* were also found to be upregulated in microglia in vivo from aged mice and demonstrated regional differences in the CNS^33^. In vitro, IRF7 was recently shown to control the activation state of microglia and responsiveness to TGF-β^34^. Deficiency in USP18, an upstream regulator of IRF7, also results in aberrant CNS interferonopathy^35,36^. Collectively, these studies suggest that IRF7 is a critical transcription factor regulating aberrant microglia activation states.

The gray matter microglia activation that occurs after depletion is consistent with previous observations of gliosis following microglia repopulation using a different CX3CR1-Cre^*ERT2*^ mouse line^18^. As compared to this study, we obtained rapid depletion after DT injection (95% of all microglia) that resulted in a large pool of cells that died over a short period of time. It is important to note that we utilized a non-physiologic tool to rapidly ablate 95% of the microglia in the entire CNS. The neurologic and inflammatory phenotype we observed is specific to the acute and synchronous nature of the ablation we used as this phenotype is not observed with CSFR1 inhibitors such as PLX5562. However, focal ablation of microglia, with subsequent rapid repopulation, has been shown to occur in instances of stroke^37^. In light of our findings, it may be relevant to investigate how microglia-regulated type 1 interferon responses modulate focal neurodegeneration in models of stroke, traumatic brain injury and viral infection.

Dying cells release a number of damage-associated molecular patterns (DAMPs), such as adenosine triphosphate (ATP) and DNA, that are known to activate downstream pattern recognition receptor signaling pathways, including Toll-like receptors and Nod-like receptors^38^. Indeed, DNA and RNA are potent inducers of IRF3 and IRF7 innate type 1 interferon response pathways downstream of cytosolic RIG-I/MDA-5 receptor signaling pathways^38^. Thus, the acute depletion of 95% of microglia in the CNS could theoretically trigger a robust nucleic acid DAMP-mediated inflammatory cascade, resulting in the global type 1 interferon response we observed in layer IV of the somatosensory cortex, which is supported by our staining of 9D5+ nucleic acids in the somatosensory cortex at d10 post depletion. Both neurons and astrocytes have been shown to secrete type I interferon during chronic EAE^39^ and in response to nucleic acid DAMPs^40^, and recent findings report that neurotoxic astrocytes can be induced by activated microglia^41^.

The precise role that microglia play in the pathogenesis of EAE is unclear. Microglia become activated during EAE and express higher levels of antigen presentation molecules such as MHC I and II and costimulatory markers such as CD80 and CD86^42^. Moreover, inhibiting microglia activation pathways have been shown to limit the severity of EAE^43^. Studies have also shown that arresting microglia cell proliferation by injecting ganciclovir in CD11b-HSV-TK-targeted mice reduced EAE pathology^44^, although other studies have demonstrated that ganciclovir treatment on its own non-specifically attenuates EAE^45^. Recent studies have proposed that infiltrating Ly6C^Hi^CCR2^+^ monocytes, and not microglia, act in concert with myelin-specific T cells to drive spinal cord pathology in EAE^46,47^. In our study, microglia were depleted prior to EAE or at a time of maximal EAE-induced CNS inflammation. Despite this, we did not observe differences in EAE-related T cell-mediated inflammation, demyelination or neurodegeneration in the spinal cord. Similarly, the EAE-related inflammatory response did not affect neurodegeneration associated with microglia repopulation and whether CD8^+^ T cells or B cells would amplify the neurodegeneration in our model is unclear^48^. Moreover, microglia depletion during active EAE led to a recruitment of Ly6C^Hi^ monocytes to the CNS, which is in line with a previous study that demonstrated increased Ly6C^Hi^CCR2^+^ monocyte recruitment to the CNS during inflamed conditions (^27–29^). In our EAE experiments, brain and spinal cord were pooled for each mouse and it would be of future interest to dissect regional differences in Ly6C^Hi^ monocyte recruitment after microglia depletion and repopulation.

Type 1 interferons have been used as a treatment for MS. Thus, it is notable that the upregulated CNS type 1 interferon response induced by microglia depletion/repopulation did not have a beneficial effect on EAE pathology. Consistent with this, a recent study described a novel type II EAE that exhibited high CNS levels of type 1 interferon and increased neuronal damage and refractoriness to type 1 interferon treatment^49^. Moreover, studies using progressive models of EAE have reported higher expression of type 1 interferon pathways during the chronic phase of disease^39^. Thus, studying type 1 interferon signaling pathways in MS could lead to a deeper understanding of MS disease subtypes^50^.

A growing number of neurological diseases are characterized by overactive type 1 interferon responses, termed interferonopathies, including Aicardi–Goutieres syndrome^51^ and Down’s syndrome^52^. Indeed, Aicardi–Goutieres syndrome is caused by mutations in nucleic acid-sensing genes^53–55^, such as TREX1 and MDA5, that result in aberrant cellular responses to RNA and DNA products^56^. More recently, defects in the gene senataxin, which is associated with ataxia and oculomotor apraxia, was demonstrated to result in a fully dysregulated innate type 1 interferon pathway^57^. Additionally, defects in RNA processing have been associated with gene signatures observed in neurodegenerative models^33^. Consistent with our results, recent studies have demonstrated links between microglia type 1 interferon and neurodegeneration. Specifically, microglia in lupus have been shown to become pathogenic in a type 1 interferon manner^58^, microglia sorted from Alzheimer disease amyloid precursor protein (APP)-transgenic mice demonstrated a type 1 interferon profile^59^ and inhibiting type 1 interferon signaling in microglia was shown to blunt age-related deficits^60^.

In conclusion, our study provides direct evidence that microglia dysfunction, type 1 interferon signaling and neurodegeneration are linked and suggest that therapeutic strategies targeting type 1 interferon responses may be of benefit in selected neurological diseases.

## Methods

### Mice

C57BL/6 wild-type, CX3CR1-Cre^ERT2^, CX3CR1-Cre^ERT2+/–^iDTR^+/–^ and littermate CX3CR1-Cre^ERT2+/–^ mice were originally purchased from Jackson Labs. Age- and sex-matched CX3CR1-Cre^ERT2+/–^iDTR^+/–^ and littermate CX3CR1-Cre^ERT2+/–^ mice were injected with TAM (Sigma) dissolved in peanut oil and mice were injected intraperitoneally (i.p.) with 10 µg of TAM for 5 consecutive days. Microglia depletion was induced by injection of 1 µg/mouse of diphtheria toxin (Sigma) for 3 consecutive days. Minocycline is tetracycline derivative that has been extensively studied to blunt neuroinflammation by inhibiting caspase-1, caspase-3 and p38 mitogen-activated protein kinase signaling pathways. Minocyline injections were performed as previously described^24^. Briefly, minocycline was injected i.p. at 50 mg per kg of body weight twice daily for the first 2 days, then once daily for the 25 mg per kg of body weight for the duration of the experiment. The CSFR1 inhibitor PLX5562 (graciously provided by Plexxikon) was prepared in the chow and administered ad libitum as described previously^21^. Anti-IFNAR antibodies and isotype (Bioxcell) were administered as previously described^58^. Briefly, 250 µg was injected i.p. 1 day prior to DT injection and every fourth day thereafter. Sample size calculations and randomization was not performed and the investigators were not blinded for in vivo experiments. Mice were housed and treated in a conventional specific pathogen-free (SPF) facility at the Harvard Institutes of Medicine (HIM) and at the Building for Transformative Medicine (BTM) SPF animal facilities according to the animal protocol with the full knowledge and permission of the Standing Committee on Animals at the HIM and the Brigham and Women’s Hospital Institutional Animal Care and Use Committee (BTM).

### Ataxia scoring

Ataxia scoring was performed according to a previously established protocol^61^. Briefly, 4 individual tests are scored on a scale of 0–3, with 0 representing no phenotype and a score of 3 being the most severe manifestation, and then aggregated to a maximal score of 12. The individual parameters are: a ledge test, hind limb clasping, gait and kyphosis. Each test was performed at least 3 times.

### Experimental autoimmune encephalomyelitis

EAE was induced by immunization with 100 µg per mouse of MOG_35–55_ peptide emulsified in CFA (Difco Laboratories) and i.p. injection of pertussis toxin (150 ng per mouse; List Biological Laboratories, Inc.) on days 0 and 2. Clinical signs of EAE were assessed according to the atypical EAE scoring system described previously^62^. Briefly, atypical EAE is scored as: 0, no disease; 1, decreased tail tone or mild balance defects; 2, hind limb weakness, partial paralysis, or severe balance defects that cause spontaneous falling over; 3, complete hind limb paralysis or very severe balance defects that prevent walking; 4, front and hind limb paralysis or inability to move body weight into a different position; 5, moribund state.

### Rotarod

Rotarod testing was performed as previously described^63^. Briefly, mice were placed on the Rotarod at a starting speed of 4 rpm with an acceleration rate of 40 rpm. Total time spent on the Rotarod was measured up to a maximum of 300 s.

### Immunohistochemistry

Mice were killed and transcardially perfused with 20 ml of ice-cold Hank's Balanced Salt Solution (HBSS). CNS tissue was removed and fixed overnight in 4% paraformaldehyde (PFA) at 4 °C and the next day the tissue was transferred to phosphate-buffered saline (PBS). Fixed paraffin tissue blocks were cut into 5 µm thick sections, which were collected on glass slides. Immunohistochemical stainings were performed against the targets NeuN (MAB377, Millipore), P2ry12, Iba1 (PAS-27436, Thermo Fisher), Mac3 (553322, Pharmingen), GFAP (Z0334, Dako), anti-myelin CNP (Smi 91, BioLegend), Oas1a (sc-365072, Santa Cruz) and 9D5 (gift by Dr Howard Lipton) TMEM119 (ab209604 Abcam). Briefly, the slides were deparaffinated with xylene and washed in 96% ethanol prior to blocking of endogenous peroxidases in methanol containing 1% H_2_O_2_. Subsequently, the slides were rehydrated in a series of descending ethanol concentrations (96%, 70%, 50%). Next, heat-induced epitope retrieval was achieved by incubation of the sections in either EDTA buffer pH 8.5 (P2ry12, Iba1, Mac3, GFAP, CNP) or citrate buffer pH 6.0 (NeuN, Oas1a) for 60 min in a household food steamer. Sections were blocked for 20 min with 10% fetal calf serum (FCS) diluted in Dako wash buffer (DAKO) and then incubated with primary antibodies diluted in FCS/DAKO overnight at 4 °C (NeuN 1:250; P2ry12 1:2500; Iba1 1:3000; Mac3 1:150; GFAP 1:3000; CNP 1:2000; Oas1a 1:100; 9D5 1:5000). The following day, sections were washed 3 times in tris-buffered saline (TBS) and appropriate biotinylated secondary antibodies diluted in 10% FCS/DAKO were incubated at room temperature for 60 min. After a TBS washing step, avidin-peroxidase was diluted 1:500 in 10% FCS/DAKO and applied for 60 min. After washing, sections were developed with 3,3'-diaminobenzidine (DAB) 1:50 in PBS supplemented with 1:300 H_2_O_2_. Counterstaining with hemalaun and Scott’s solution was performed to stain the cell nuclei blue. Finally, the slides were dehydrated in an ascending ethanol series and mounted with Eukitt. Manual counting was done with a morphometric grid in the eyepiece using the 40× objective of a conventional light microscope (Olympus BX50). Cell numbers were determined within six microscopic fields in motor cortex regions, sensory cortex regions and spinal cord. The number of cells per grid was calculated to cells per mm^2^. For digital optical densitometry, high-resolution images (2560 × 1920 pixels; 1.52 × 1.14 mm to cover the entire depth of the cortex) of immunohistocehmical stainings were obtained in motor and somatosensory cortical regions using a Nikon DS-Fi1® digital camera mounted on a Reichert Polyvar 2® microscope with the 4× objective using the Nikon NIS Elements® software version 3.10. Images were acquired under standardized conditions by controlling the lamp brightness and camera white balance before each imaging session when no slide was under the objective. The area fraction (AF) of the stainings was determined using ImageJ® version 1.43r (16). To separate blue hemalaun counterstaining from brown DAB staining, a color deconvolution plug-in (freeware kindly provided by A.C. Ruifrok, NIH) was run using the vector H-DAB. Resulting red channels containing the dissected DAB signal were converted into 8-bit gray scale images. To assess the AF of the stainings, the mean gray value of each image was subtracted from every pixel in that image for background removal. Subsequently, a threshold of 60/255 was set, resulting in a black and white matrix, with black pixels reflecting cellular staining. The AF was determined as percentage of black pixels in the images.

### Confocal imaging

Mice were killed and transcardially perfused with 20 ml of ice-cold HBSS. CNS tissue was removed and fixed overnight in 4% PFA at 4 °C. The next day the brains were transferred to 30% sucrose solution and then flash frozen on dry-ice and stored at −80 °C until sectioning. Then, 10 µm sections were blocked in 5% goat serum, M.O.M.™ Mouse Ig Blocking reagent (Vector laboratories) containing 0.3% Triton™ X-100 (Sigma-Aldrich), and incubated overnight at 4 °C with following antibodies: GFAP (chicken, 1:500, Abcam), IBA-1 (rabbit, 1:200, Dako), Nestin (mouse (rat-401), Millipore). The next day, sections were washed 3 times, and incubated with an appropriate fluorophore-conjugated goat secondary antibodies (1:1000; Abcam) for 1 h at room temperature. Six animals per group were used. Images were taken using a LSM 710 Zeiss confocal microscope.

### Flow cytometry and cell sorting

These purification procedures are based on previously described dissociation and purification protocols^10^. Isolated CNS cells were incubated with anti-mouse CD16/CD32 for 15 min on ice to block the Fc receptors, and stained with fluorochrome-conjugated antibody for CD11b (M1/70), CD45(90), CD3 (145-2C11), CD4 (GK1.5) and Ly6C (HK1.4). Microglia were sorted on a BD FACS Aria II as CX3CR1-GFP^+^CD11b^+^ cells with low CD45 and low Ly6C expression (CX3CR1^+^CD11b^+^/CD45^low^/Ly6C^low)^.

### Intracellular FACS staining

For intracellular cytokine staining, cells were stimulated for 6 h with phorbol 12-myristate 13-acetate (50 ng per ml; Sigma), ionomycin (1 µg per ml; Sigma) and monensin (GolgiStop; 1 μl per ml; BD Biosciences). After staining of surface markers, cells were fixed and permeabilized according to the Foxp3 Fixation/Permeabilization (Ebioscience) manufacturer’s instructions.

### Mass cytometry (CyTOF)

A single suspension of mouse non-parenchymal cells were stained with the viability marker cisplatin, then blocked with rat-anti mouse CD16/32 (BD clone 2.4G2) and stained with the metal-coupled antibody panel described in Suppl. table 1. The next day, cells were washed and incubated with DNA-Intercalator solution, then washed in MilliQ water (low barium) and resuspended with 1:10 dilution of EQ beads (for normalization). Samples were acquired and normalized on a CyTOF2 (Fluidigm, Inc.) running the Helios platform. Normalized data were imported to Cytobank, where gating was performed as described in Supplementary Fig. 5 and ViSNE analysis was performed as described previously^64^.

### RNA-sequencing

RNA from cortical tissue was isolated using Trizol extraction as previously described^65^. Total RNA samples were QC’d by Agilent Bioanalyzer for RNA Integrity Scores (RIN > 8), and normalized by Nanodrop to a minimum of 5 ng/μl and 250 ng. Libraries were constructed using Illumina’s TruSeq kit with Poly A selection, pooled and sequenced on the Illumina NextSEQ with 75 bp paired-end reads to a read coverage of 30 million reads per sample. RNA-seq reads were aligned using Tophat^66^ and RSEM-based quantification using known transcripts performed^67^, followed by further processing using the Bioconductor package DESeq in R^68^. The data were normalized using the trimmed mean of M-values (TMM) normalization, a simple and effective method for estimating relative RNA production levels from RNA-seq data^67^. The TMM method estimates scale factors between samples that can be incorporated into currently used statistical methods for DE analysis. Post-processing and statistical analysis was carried out in R^67^. DE genes were defined using the differential expression pipeline on the raw counts with a single call to the function DESeq^68^ (adjusted *p* value < 0.1).

### Nanostring analysis

RNA from sorted microglia was isolated using mirVana RNA isolation kits (Thermo Fisher) according to the manufacturer’s protocol. Then, 50–100 ng of total RNA was hybridized with reporter and capture probes for nCounter Gene Expression code sets (Mouse Immunology Codeset) according to the manufacturer’s instructions (NanoString Technologies). Data were normalized to spiked positive controls and housekeeping genes (nSolver analysis system). Transcript counts less than the mean of the negative control transcripts plus 2 standard deviations for each sample were considered background.

### Ingenuity

Data were analyzed through the use of IPA (Ingenuity® Systems). Differentially expressed genes (with corresponding fold-changes and *p* values) were incorporated in canonical pathways and bio-functions and were used to generate biological networks.

### T-cell thymidine incorporation

Splenocytes were cultured in X-VIVO medium and were plated for 72 h at a density of 5 × 10^5^ cells per well in the presence of MOG_35–55_ peptide. During the final 16 h, cells were pulsed with 1 Ci [3 H]thymidine (PerkinElmer) followed by collection on glass fiber filters and analysis of incorporated [3H]thymidine using a counter (1450 MicroBeta TriLux; PerkinElmer).

### Quantitative real-time reverse transcription–polymerase chain reaction (qRT-PCR)

EXPRESS One-Step SuperScript qRT-PCR kit (Thermo) was used according to the manufacturer’s protocol for qPCR analysis. TaqMan probes (Thermo) were used for *oas1a* (Mm00836412_m1) and *mx1 (*Mm00487796_m1).

### Laser microdissection

Fixed tissue blocks of paraffin embedded tissue were cut into 8 µm thick sections, which were collected on membrane slides and dried with desiccant under RNase-free conditions at room temperature for a minimum of 3 days. Prior to laser microdissection, the brain sections were dewaxed by two incubations with xylene (each for 2 min) followed by a washing step with iso-propanol for 45 s. The tissue sections were covered for 30 s with staining solution (hematoxylin and eosin (H&E) Staining Kit Plus, Molecular Machines and Industries, MMI), followed by an immediate stop of the reaction by decanting the stain and washing the slides in nuclease-free water for 30 s. After additional washing steps with iso-propanol (45 s) and xylene (twice for 2 min each), the slides were briefly air-dried. Using an inverted light microscope (Nikon Eclipse Ti) equipped with a CellCut laser microdissection system (MMI), regions of interest (somatosensory and motor cortex) were marked via CellTools v4.4 software (MMI) and cut out as quickly as possible to ensure sufficient RNA quality. Cutting was performed with a ultraviolet laser and tissues from different cortical areas were collected in separate isolation tubes. RNA was isolated from the laser-assisted microdissected tissue using the High Pure formalin-fixed, paraffin-embedded (FFPE) RNA Micro Kit (Roche) according to the manufacturer’s protocol. To determine RNA quality and quantity, 1 µl total RNA was loaded on Agilent RNA Pico Chips and analyzed using an Agilent 2100 Bioanalyzer. For each FFPE-derived sample, the electropherogram was plotted and the DV200 was calculated using 2100 Expert software.

### Microarray of laser-microdissected tissue

RNA (2 ng) isolated from laser-assisted microdissected tissue (somatosensory as well as motor cortex derived from four control and four d10 microglia-depleted mice) was transcribed, amplified and labeled using the GeneChip WT Pico Reagent Kit (Affymetrix) according to the manufacturer’s instructions and subsequently hybridized to a GeneChip® Mouse Gene 2.1 ST 16-Array Plate (Affymetrix) according to the manufacturer’s protocol. For microarray processing (washing, hybridization, signal detection), a GeneTitan® instrument (Affymetrix) was used. The raw data were processed by the Affymetrix Gene Chip Command Console (AGCC) and normalization was performed using the Affymetrix Expression Console (EC). Additionally, quality control was done with the EC by calculating several metrics (AUC, PM-mean, BG-mean, MAD, RLE, spike-ins). After quantile normalization, the data were exported to CHP files, which can be read by the Affymetrix Transcriptome Analysis Console (TAC).

### Statistical analysis

Prism GraphPad software was used for statistical analysis; unpaired two-tailed *t*-test was used for comparing two groups, one-way analysis of variance (ANOVA) with Tukey’s post-hoc test for multiple group comparisons and log-rank test for survival curves. The *p* values of less than 0.05 at 95% confidence intervals were considered significant.

## Electronic supplementary material

Supplementary Information

Peer Review File

## Data Availability

The datasets included in this study are available from the corresponding author upon reasonable request. The RNA-Seq and microarray data has been deposited in GEO (accession numbers: GSE104634 for microarray, GSE115447 for RNA-Seq).
