# Supplementary material for: Acute microglia ablation induces neurodegeneration in the somatosensory system
Source: Nat Commun. 2018 Nov 1;9:4578. doi: 10.1038/s41467-018-05929-4 (PMC6212411; doi:10.1038/s41467-018-05929-4)
Supplement: Supplementary file 1 — Supplementary Information [file 41467_2018_5929_MOESM1_ESM.pdf]

Supplementary Information

Acute microglia ablation induces type 1 interferon-driven neurodegeneration in the somatosensory system

Rubino et al.

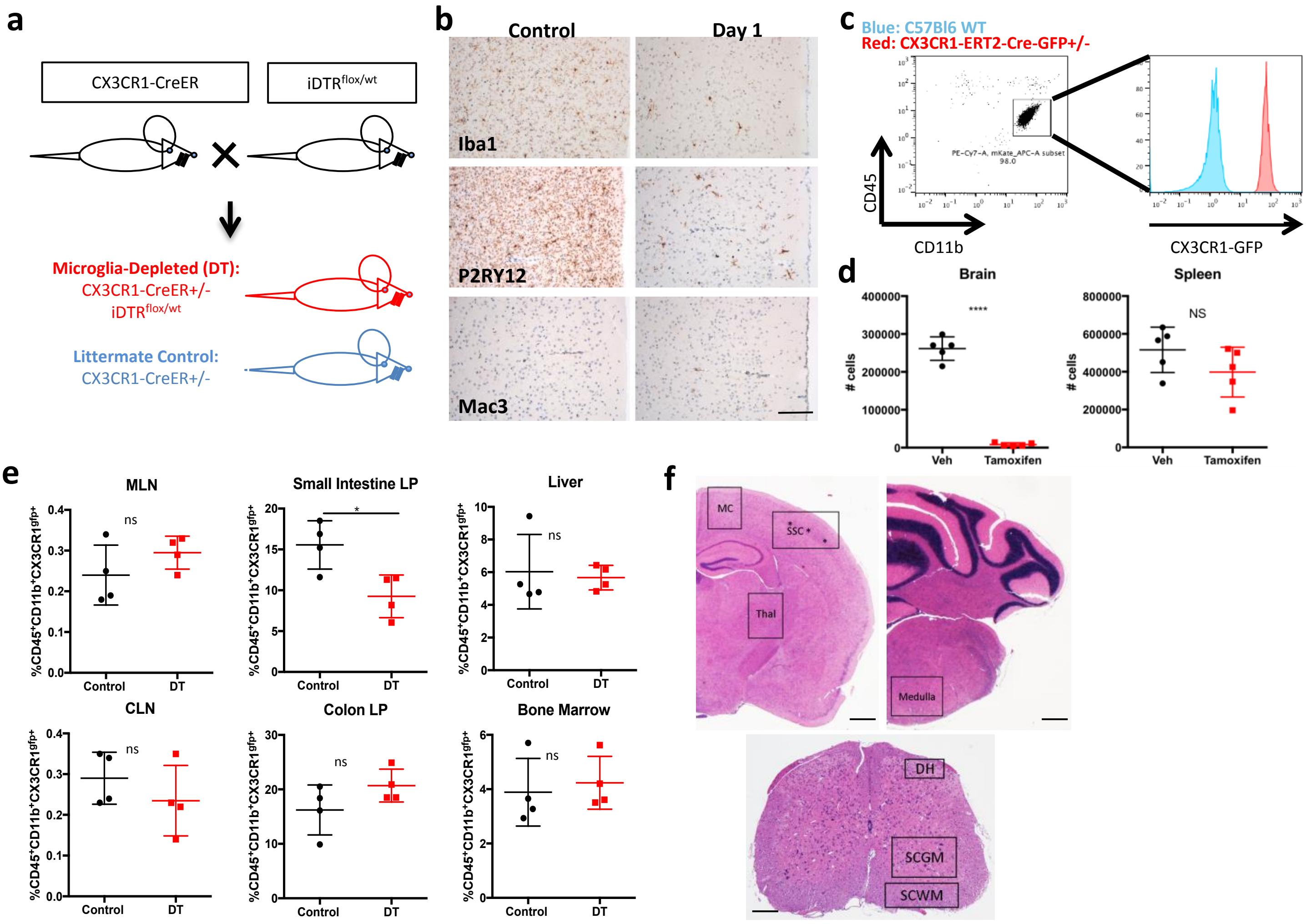

Supplementary Figure 1. Control staining of microglia depletion using CX3CR1-CreER-iDTR mice. (**a**) Schematic of mating scheme to generate CX3CR1-CreER-iDTR mice that will express the diphtheria toxin (DT) receptor under the control of a TAM-inducible Cre in the CX3CR1 locus. (**b**) Images depict immunohistochemical staining of Iba1, P2RY12 and Mac3. (**c**) Histogram depicts the expression of CX3CR1-GFP in CD11b^{+}CD45^{int} microglia from either littermate control (blue) or CX3CR1-CreER-iDTR (red) mice. (**d**) Bar graphs depict the average number of CD11b^{+}CD45^{int}CX3CR1-GFP cells at day 1 post depletion in the brain and spleen of vehicle- and TAM-treated CX3CR1-CreER-iDTR mice. (**e**) Bar graphs depict the % of CD45^{int}CD11b^{+}CX3CR1-GFP cells at day 1 post depletion in the mesenteric (MLN) and cervical lymph nodes (CLN), small intestine and colon lamina propria (LP), liver and bone marrow of CX3CR1-CreER$^{+/-}$ mice (Control) and CX3CR1-CreER$^{+/-}$iDTR$^{flox/wt}$ (DT) mice determined by flow cytometry. Representative experiment of at least two independent experiments. Error bars depict SEM and n=4-7 mice/group. Analyzed with Student's unpaired t-test at 95% confidence interval where * represents p-value < 0.05, **** represents p-value < 0.0001, NS= not significant. (**f**) Images depict H&E staind of CNS with representative regions highlighted: MC = motor cortex, SSC = somatosensory cortex, Thal = Thalamus, Medulla, DH = dorsal horn, SCGM = spinal cord grey matter, SCWM = spinal cord white matter. Scale bar for (**b**) = 100µm and scale bar for (**f**) = 200µm.

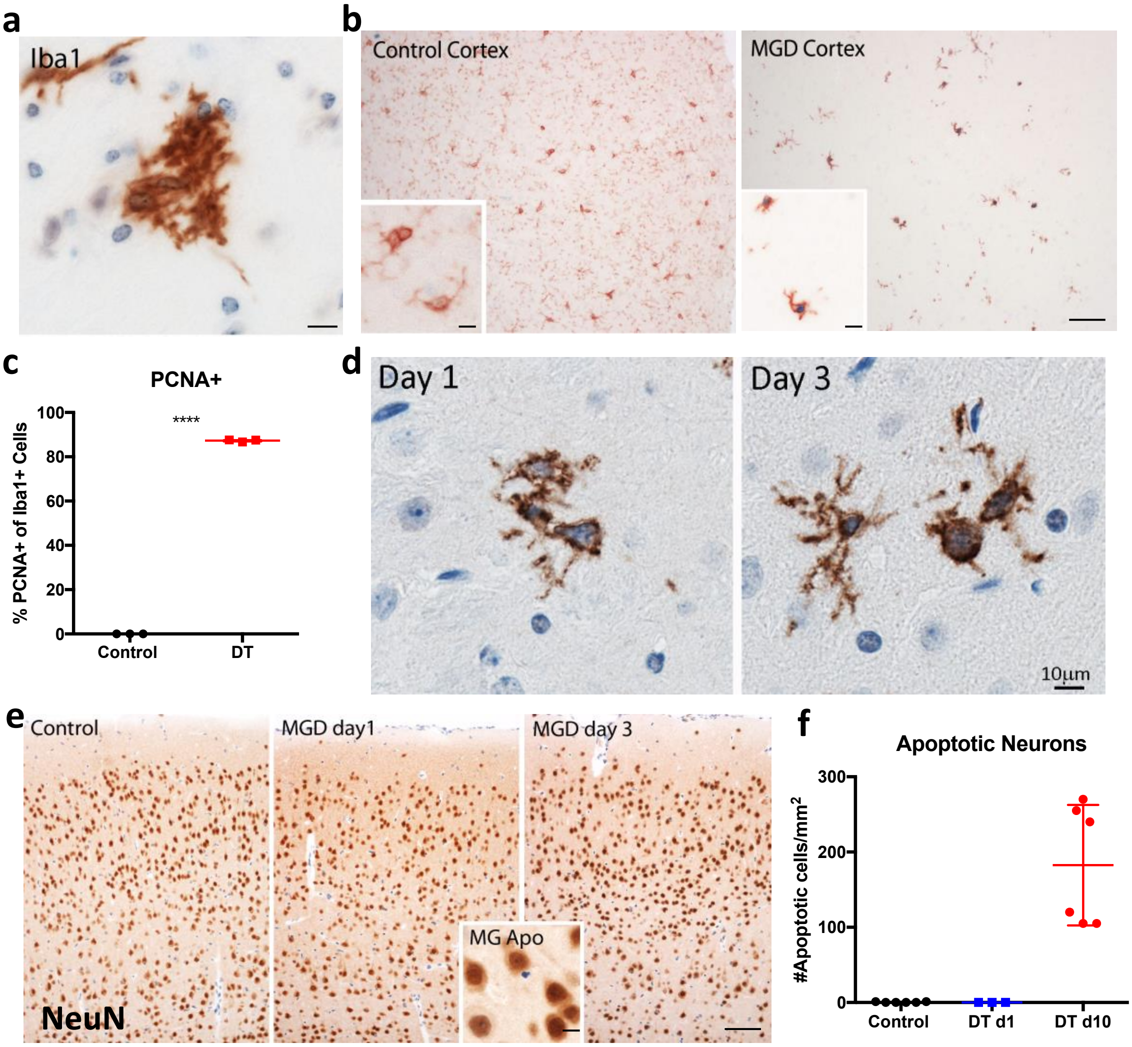

Supplementary Figure 2. Iba1, TMEM119 and NeuN staining at days 1 and 3 post depletion. (**a**) Representative staining of Iba1⁺ clusters of microglia at day 3 post depletion. (**b**) PCNA⁺ (Proliferating Cell Nuclear Antigen: a nuclear marker that is only expressed during the DNA synthesis stage of dividing cells) staining of proliferating Iba1⁺ microglia at d3 post depletion in somatosensory cortex (MGD Cortex) compared to control cortex. (**c**) Bar graph depicts %PCNA⁺ proliferating Iba1⁺ cells at day 3 post-depletion quantified by blinded manual counting of immunohistochemistry stainings. (**d**) Representative immunohistochemistry of the microglia specific marker TMEM119 of proliferating microglia cluster of day 1 post microglia depletion ("MGD" = Microglia Depleted day 1) and day 3 post depletion (MGD day 3). (**d**) Representative immunohistochemistry of NeuN neuronal staining in the somatosensory cortex of control, day 1 post microglia depletion (MGD day 1) and day 3 post depletion (MGD day 3). No loss of neurons and no neurons with apoptotic nuclei are seen. However, the insert shows the dark blue condensated apoptotic nucleus of a perineuronal microglia cell. Scale bar for (**b**) and (**e**) = 100μm, scale bar for (**a**), (**d**) and cropped (**b**) and (**e**) = 10μm. (**f**) Bar graph depicts the number of apoptotic neuronal cells at days 1 and 10 post-depletion quantified by blinded manual counting. Representative experiment of two independent experiments. Error bars depict SEM and n= 3-6 mice/group. Analyzed with Student's unpaired t-test at 95% confidence interval where p-value ****< 0.0001.

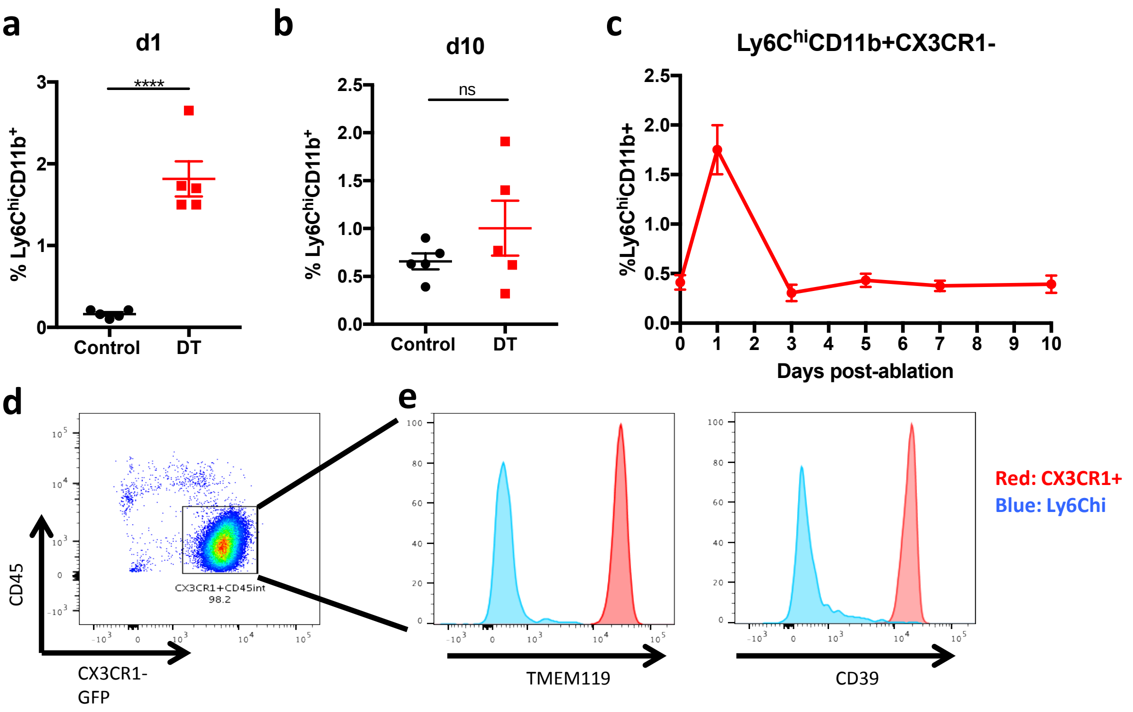

Supplementary Figure 3. Kinetics of CNS Ly6C^{hi} monocyte recruitment post-microglia depletion. a-b) Bar graphs depict % of Ly6C^{Hi}CD11b+ monocytes infiltrating the CNS at d1 (**a**) and d10 (**b**) post depletion determined by flow cytometry of whole CNS. (**c**) Time course of % of Ly6C^{Hi}CD11b+ monocytes infiltrating the CNS from at d1, d3, d5, d7 and d10 post-depletion analyzed by flow cytometry. Error bars depict SEM and n= 3-6 mice/group. Analyzed with Student's unpaired t-test at 95% confidence interval where p-value ****< 0.0001, ns = not significant. (**d**) Flow cytometry dot plot of d10 repopulated CD45intCX3CR1gfp+CD11b+ microglia, CD45 (y-axis) and CX3CR1-gfp (x-axis). (**e**) Histograms depict the expression of the microglia markers TMEM119 and CD39 on repopulated CD45^{int}CX3CR1gfp+CD11b+ microglia (red) versus Ly6C^{hi}CD11b+ infiltrating monocytes.

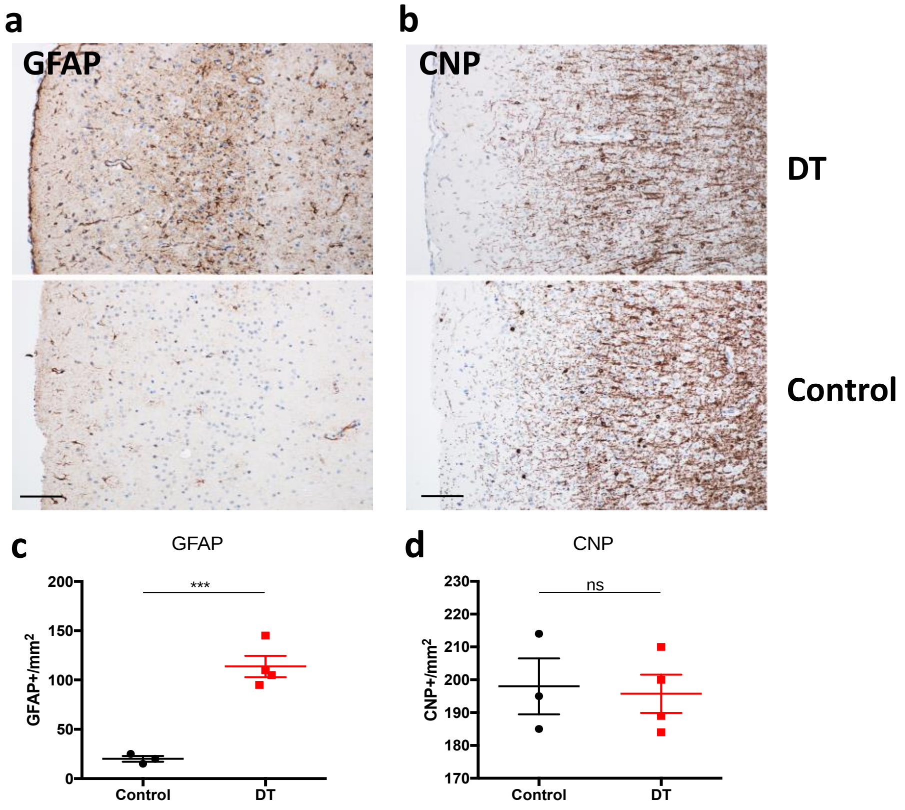

Supplementary Figure 4. GFAP and CNP staining in somatosensory cortex. (a,b) Representative immunohistochemistry staining for CNP and GFAP in the somatosensory cortex in control and d10 post depletion mice, scale bar = 100µm. (**c,d**) Bar graph depicts the number of GFAP+ cells (**c**) or CNP + cells (**d**) at d10 post-depletion quantified by blinded manual counting. Error bars depict SEM and n= 3-4 mice/group. Analyzed with Student's unpaired t-test at 95% confidence interval where p-value ***< 0.001, ns = not significant. Scale bar = 100µm.

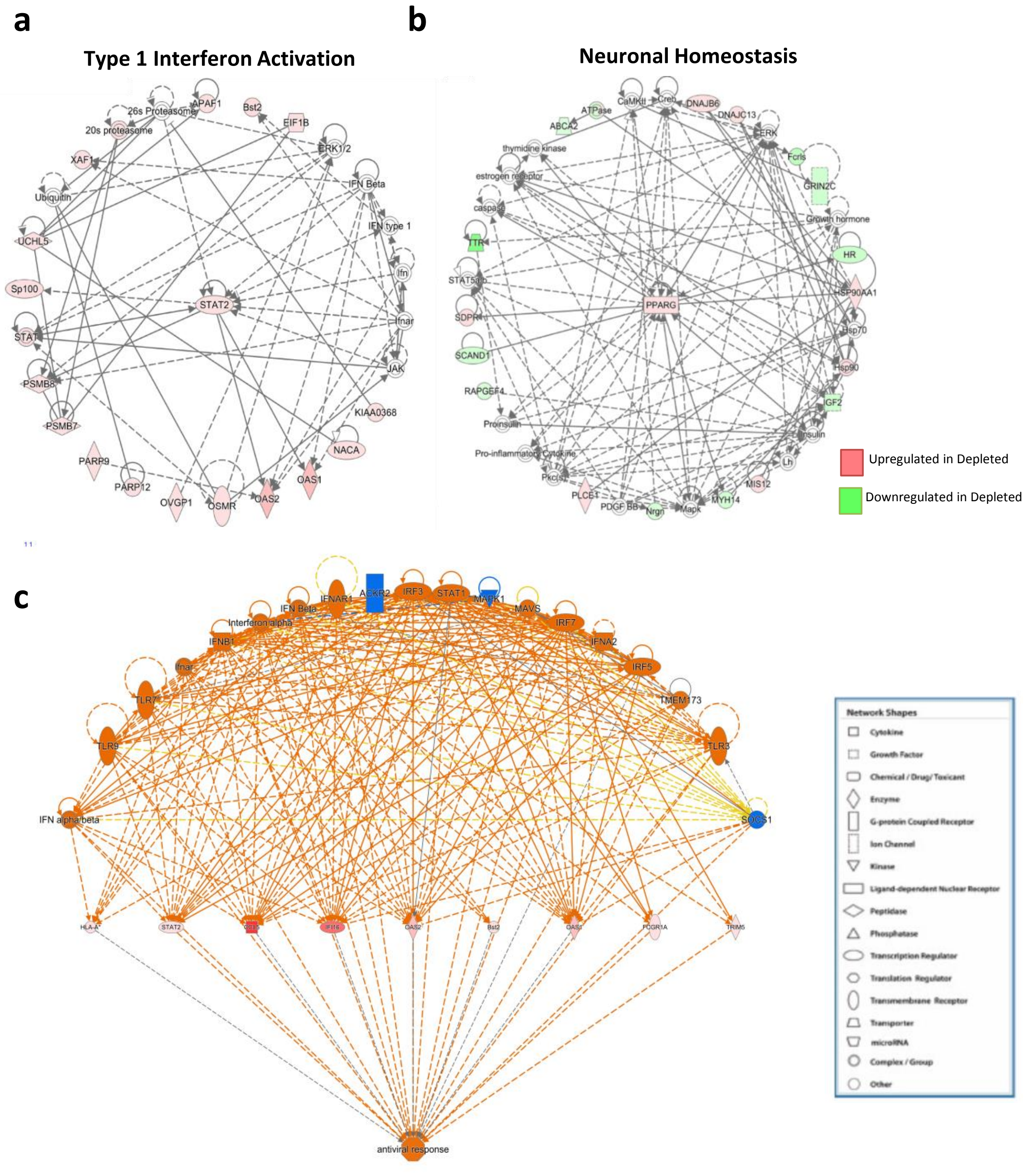

Supplementary Figure 5. Ingenuity signaling network. (a,b) Ingenuity pathway analysis identified type 1 interferon activation (a) and PPAR-g-mediated loss of neuronal homeostasis (b) biological networks in our dataset; red = upregulated in dataset, green = downregulated full lines = direct relationship, dashed lines = indirect relationship. (c) Anti-viral signaling biological network predicted to be activated in our dataset, brown = upregulated expression, blue = downregulated expression.

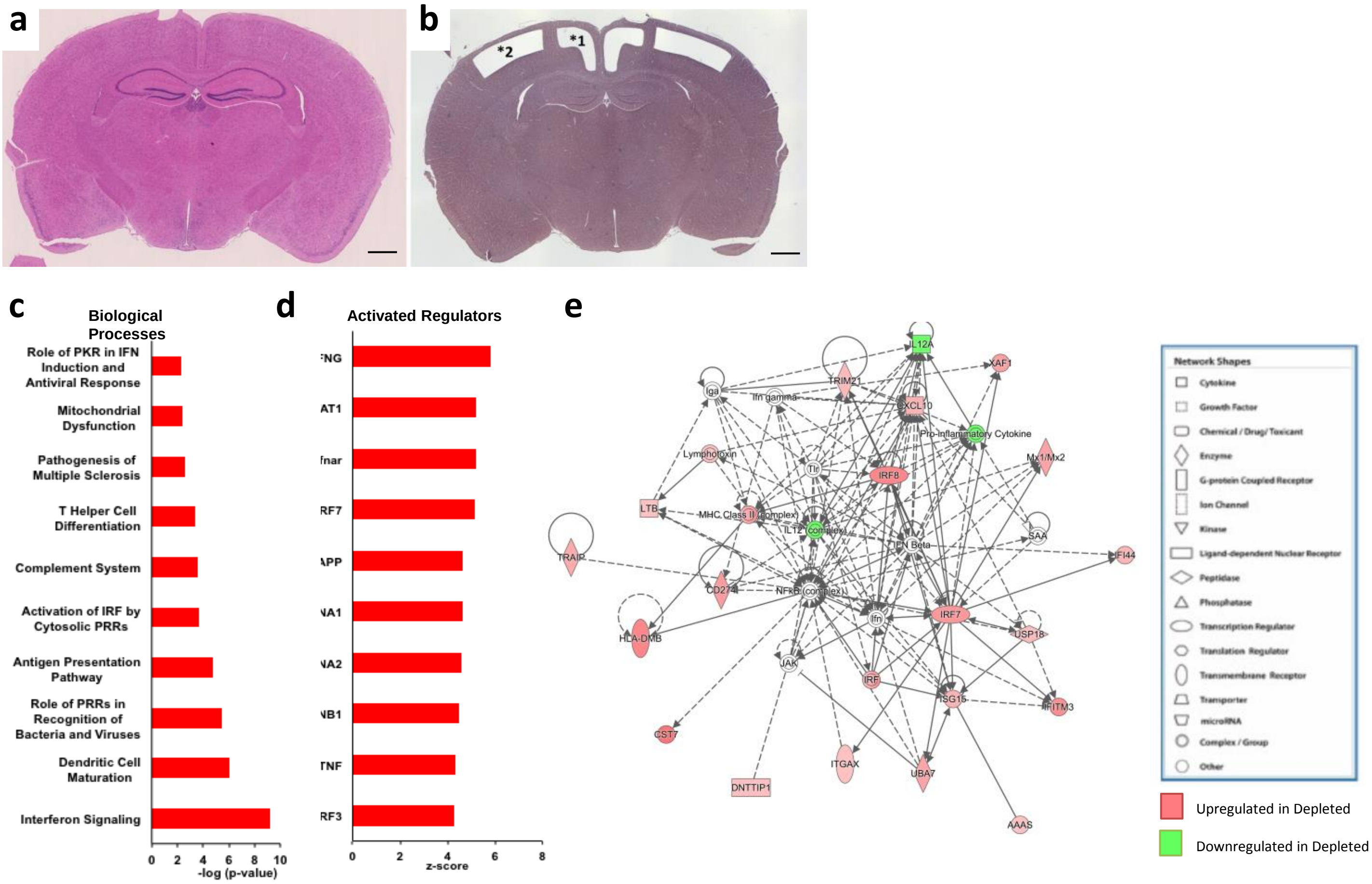

Supplementary Figure 6. Microarray analysis of microdissected layer IV somatosensory cortex. (**a-b**) Representative H&E staining of brains depicting the cortical regions microdissected for transcriptomic analysis. *1 represent the motor cortex and *2 the layer IV of the somatosensory cortex. (**c-d**) Bar graphs depict Ingenuity pathway analysis of the ten most significant biological processes (**c**) and predicted upstream regulators (**d**) of the differentially expressed genes in the dataset. (**e**) Ingenuity pathway analysis identified type 1 interferon activation biological network; red = upregulated in dataset, green = downregulated, full lines = direct relationship, dashed lines = indirect relationship. Scale bar = 200μm.

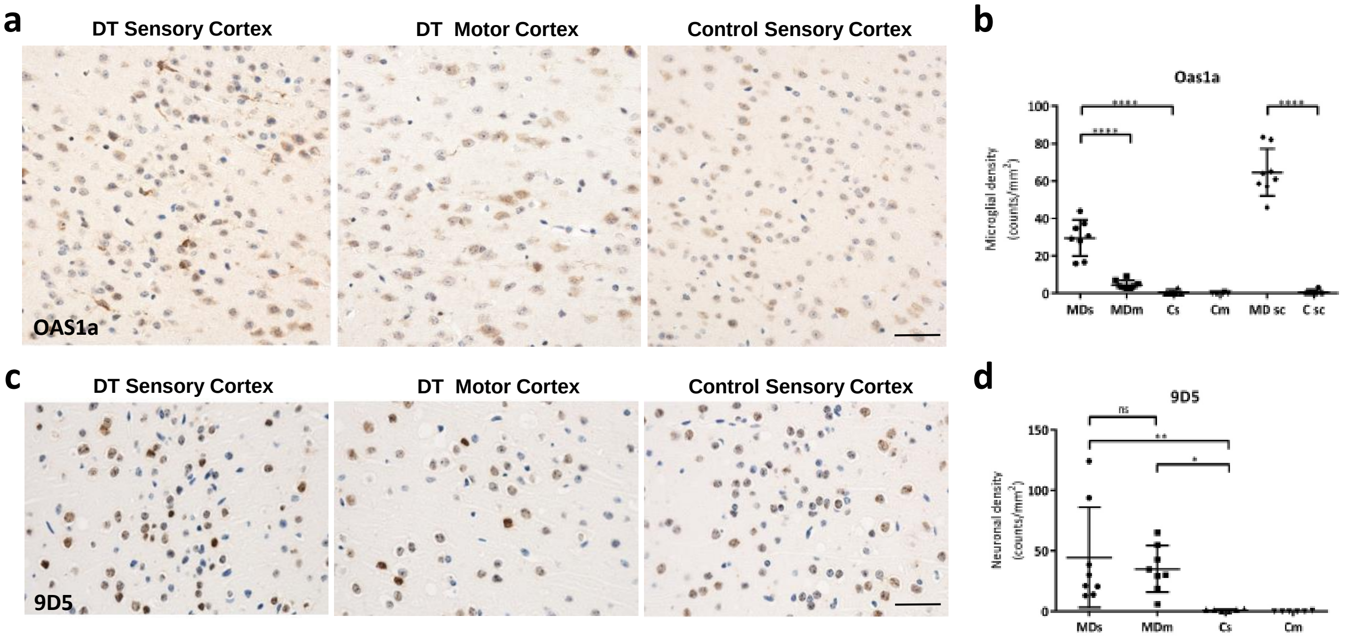

Supplementary Figure 7. Immunohistochemistry of OAS1A and dsRNA in somatosensory and motor cortex. (**a**) Representative immunohistochemistry of OAS1astaining in the sensory cortex of d10 post depletion mice (right panel), motor cortex from d10 post depletion (center panel) and sensory cortex from control animals (left panel) showing staining of microglia in the areas of degeneration, but not in the motor cortex of microglia-depleted animals or in the sensory cortex of control animals. (**b**) Scatter plot depicts quantification of $OAS1a^+$ microglia per mm^2 for microglia-depleted sensory cortex at day 10 (MDs), microglia-depleted motor cortex (MDm), control sensory cortex (Cs), control motor cortex (Cm), microglia-depleted spinal cord (MD sc) and control spinal cord (C sc). (**c**) Representative immunohistochemistry of dsRNA (9D5 on graph) staining in the somatosensory cortex of d10 post-depletion (right panel), motor cortex from d10 post-depletion (center panel) and control sensory cortex (left panel). (**d**) Scatter plot depicts quantification of 9D5+ neurons per mm^2 in the sensory (MDs) and motor cortex (MDm) of microglia-depleted mice, the sensory (Cs) and motor cortex (Cm) of control animals. Error bars depict SD and n=5-8 mice/group. Statistical calculations were done with one-way ANOVA followed by Sidak multiple comparisons correction. ****: $p<0.0001$, ***: $p<0.001$, **= $p<0.01$, * p-value < 0.05 and NS= not significant. Scale bar = 100μm.

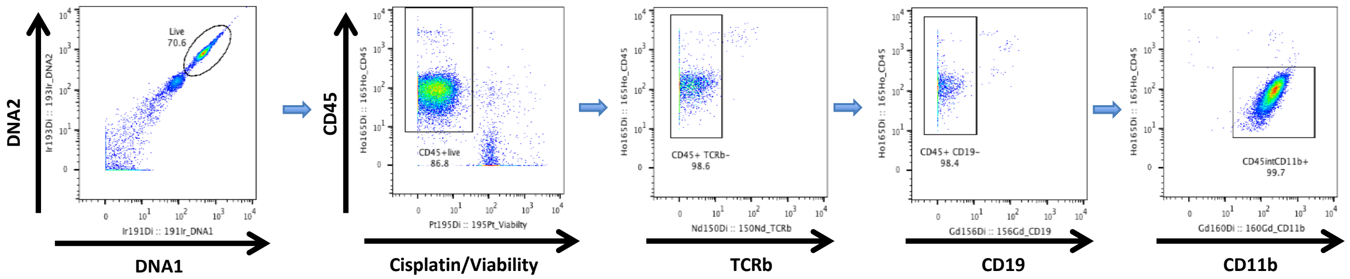

Supplementary Figure 8. Gating of microglia for CyTOF analysis. Dot plots depict the gating strategy to isolate $CD45^{int}CD11b^{+}CD3^{-}CD19^{-}$ microglia for viSNE analysis. Cells were gated based on DNA1 and DNA2 double-positive cells. Viable leukocytes were gated as $CD45^{+}$ and cisplatin-negative. Microglia were gated as $CD45^{int}$ and $CD11b^{+}$ cells on $CD19^{-}$ and $TCRb^{-}$ gated cells. One representative of two independent experiments shown.

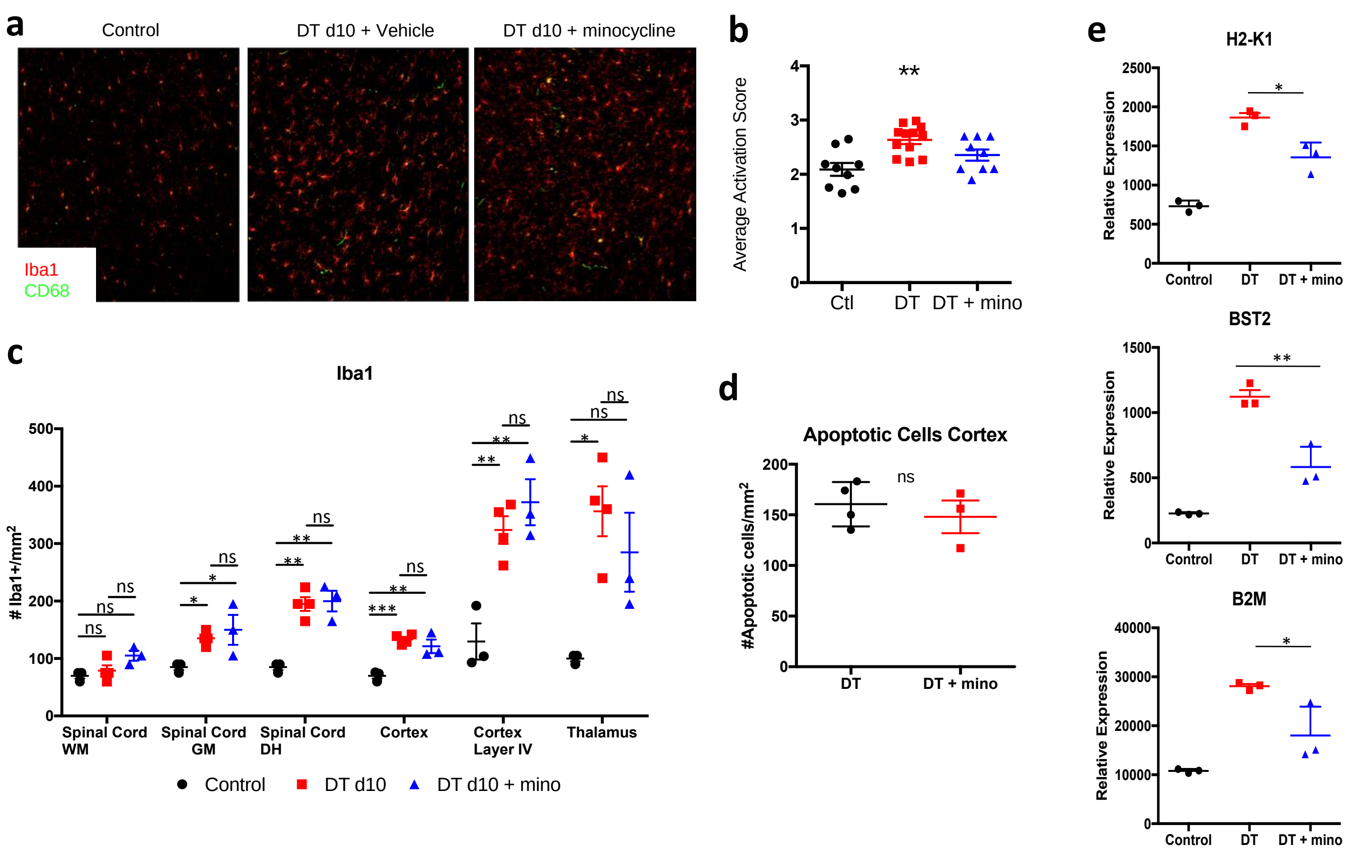

Supplementary Figure 9. Impact of minocycline treatment on repopulated microglia. (**a**) Confocal images depict Iba1 (red) and CD68 (green) staining in the somatosensory cortex of controls, microglia-depleted mice and microglia-depleted animals treated with minocycline at d10 post depletion. (**b**) Dot plot depicts quantification of confocal CD68^{+} activation of control (Ctl), microglia-depleted (DT) and microglia-depleted mice treated with minocycline (DT + mino). CD68 is a scavenger receptor expressed in the cytosol that is upregulated on myeloid cells when they become activated. Error bars depict SEM and n=9-13 mice/group. Analyzed with one-way ANOVA. (**c**) Dot plots depict the average number of Iba1^{+} cells per mm^{2} quantified in control (black), d10 post microglia depletion (red) and d10 post-depletion injected with minocycline (blue) in spinal cord grey matter (GM), spinal cord white matter (WM), the frontal cortex and the somatosensory system, including the dorsal horn of the spinal cord, the thalamus, the layer IV of the somatosensory cortex. Analyzed by one-way ANOVA with Tukey' post-hoc test for multiple comparisons. (**d**) Dot plots depicts quantification of number of apoptotic cells/mm^{2} in layer IV somatosensory cortex of d10 post depletion mice (black) and d10 post depletion + minocycline (red). (**e**) Dot plots demonstrate the relative expression levels of *H2-K1*, *Bst2* and *B2M* in control (black), d10 post-depletion microglia (red) and d10 microglia-depleted mice treated with minocycline (blue). Representative experiment of two independent experiments. Error bars depict SEM and n=3-5 mice group. (d-e) Analyzed with student's unpaired t-test at 95% confidence interval. ****: $p<0.0001$, ***: $p<0.001$, **= $p<0.01$, * p-value < 0.05 and NS= not significant.

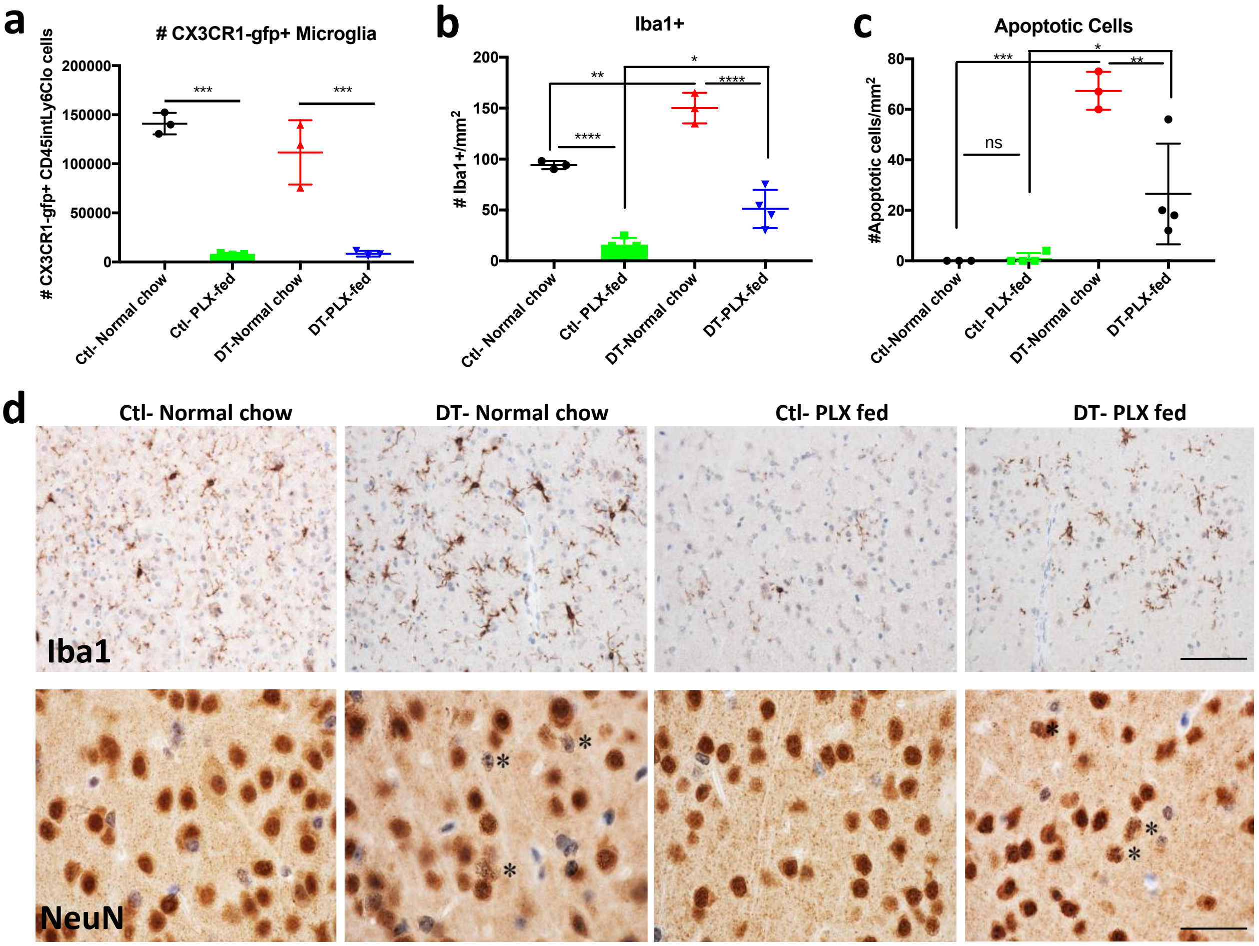

Supplementary Figure 10. Iba 1 and NeuN staining in PLX5562 fed animals post-microglia ablation. (**a**) Dot plot depicts the numbers of $CD45^{int}CD11b^{+}$CX3CR1-GFP cells from control mice fed control chow (black), control mice fed PLX5562 chow (green), d10 post-depletion fed normal chow (red) and d10 post-depletion fed PLX chow (blue). (**b,c**) Quantification of the number of Iba1+ cells/mm^2 and apoptotic Neurons/mm^2 in the somatosensory cortex of control mice fed control chow (black), control mice fed PLX5562 chow (green), d10 post-depletion fed normal chow (red) and d10 post-depletion fed PLX chow (blue). Analyzed with one-way ANOVA at with Tukey' post-test for multiple comparisons. ****: p<0.0001, ***: p<0.001, **= p<0.01, * p-value < 0.05 and NS= not significant. (**d**) Representative immunohistochemistry of Iba1 and NeuN in the somatosensory cortex. Scale bar = 100μm.

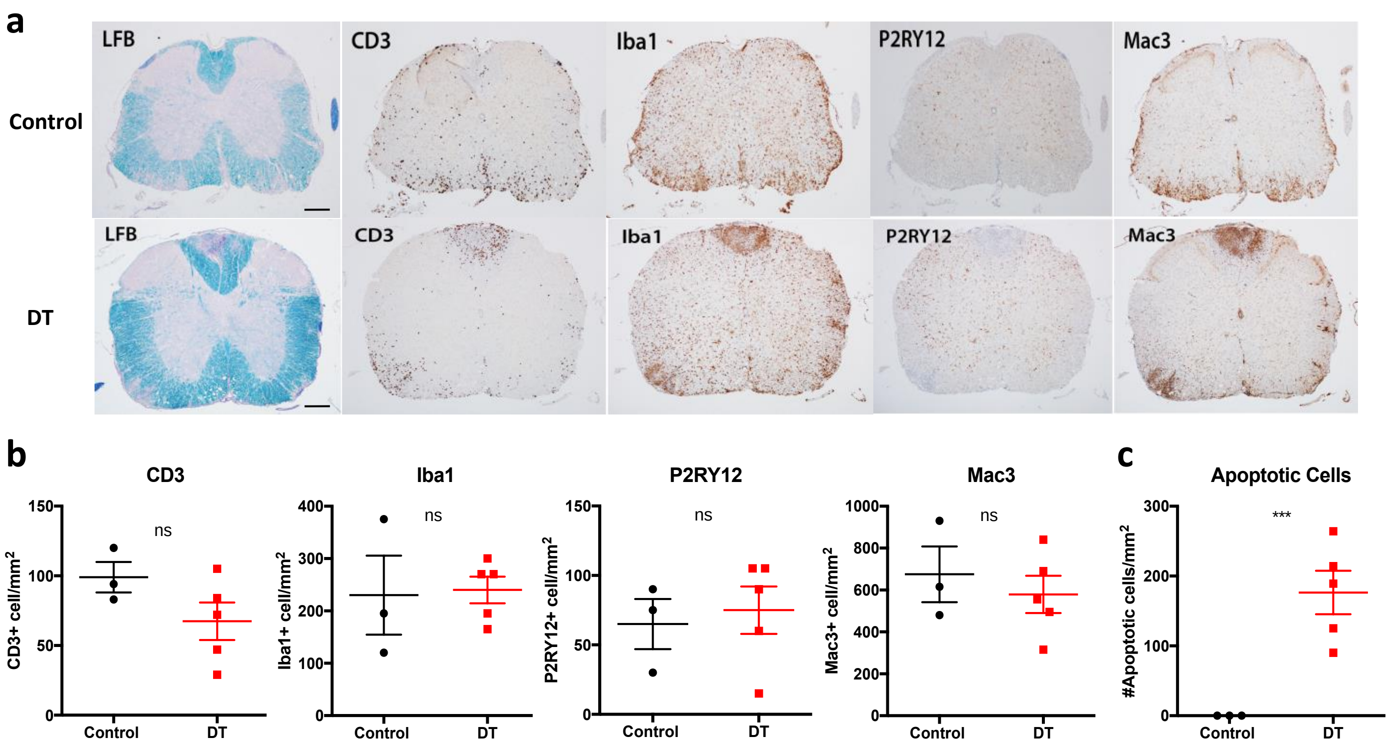

Supplementary Figure 11. CNS immunohistochemistry of microglia depletion before EAE. (**a**) Representative immunohistochemistry of Luxol Fast Blue, CD3, Iba1, P2RY12 and Mac3 staining in spinal cord (SC) of d21 EAE from control and mice depleted before EAE onset. (**b**) Quantification of the number of CD3, Iba1, P2RY12 and Mac3 cells/mm^2 in spinal cord of d21 EAE from control and mice depleted before EAE onset (DT). (**c**) Quantification of the number of apoptotic neurons/mm^2 in somatosensory cortex at d10 EAE from control and mice depleted before EAE onset (DT). Error bars depict SEM and n=3-5 mice/group. ***: $p<0.001$and NS= not significant. Scale bar = 200μm.

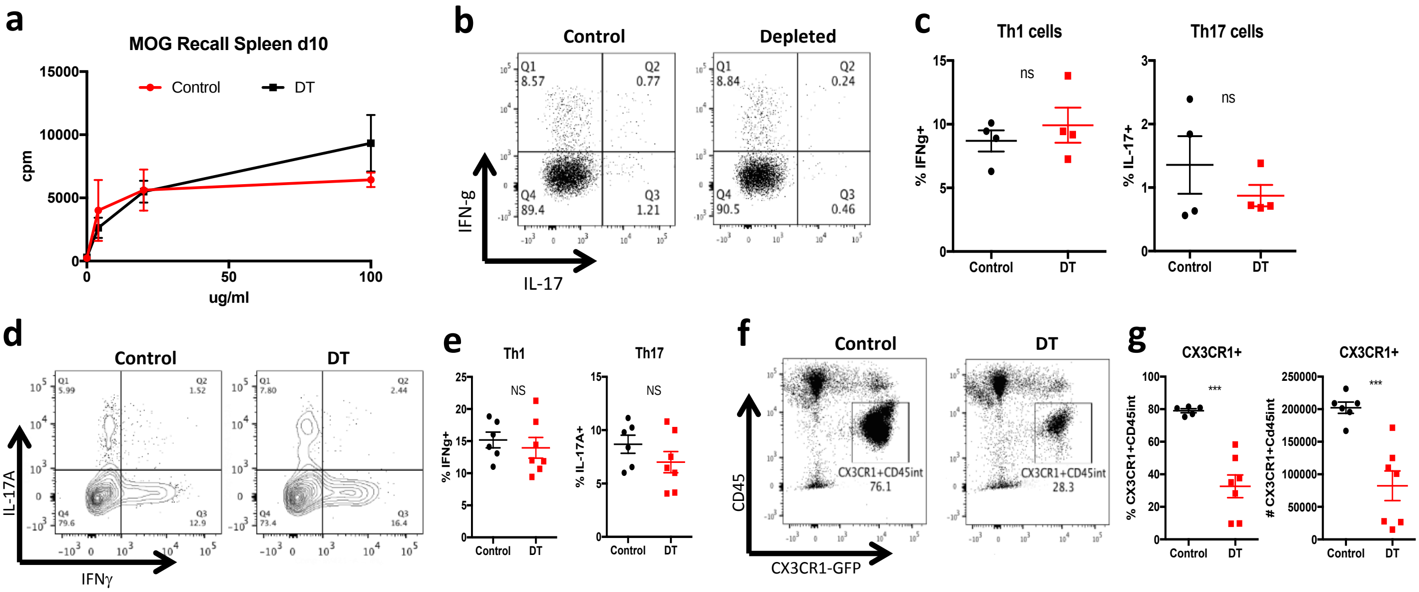

Supplementary Figure 12. T cell and myeloid cell dynamics of microglia depletion during EAE. (**a**) Recall assay depicts quantification of thymidine incorporation of T cells from control and microglia-depleted mice stimulated with MOG ex vivo. (**b**) Dot plots of IL-17A^{+} and IFN-g^{+} T cells isolated from the spleen at d10 EAE of controls and mice microglia-depleted at d0 (DT). (**c**) Quantification of percentage of Th17 and Th1 cells in spleen of control and d0 DT mice. (**d**) Contour plots of IL-17A^{+} and IFN-g^{+} T cells isolated from the SC at d35 EAE of control and microglia depleted at d21 mice (DT). (**e**) Quantification of percentage of Th17 and Th1 cells in SC control (black) and d21 microglia depleted (DT) mice (red). (**f**) Dot plot of CX3CR1-gfp and CD45 of cells isolated from brain and gated on CX3CR1-gfp^{+}CD45^{int} microglia at d35 EAE in control and depleted mice. (**g**) Quantification of percentage and number of CX3CR1-gfp^{+}CD45^{int} microglia SC control (black) and d21 microglia depleted (DT) mice (red). Error bars depict SEM and n=4-8 mice/group. ***: p<0.001and NS= not significant.

ANTIGEN	CLONE	METAL-CONJUGATE
GR1	*RB6-8C5*	*139La*
CD44	*IM7*	*141Pr*
CD115	*AFS98*	*142Nd*
TER-119	*TER-119*	*143Nd*
CD1d	*K253*	*144Nd*
CD4	*RM4-5*	*145Nd*
CD11c	*N418*	*146Nd*
CD317/PDCA-1	*927*	*148Nd*
CD274 (PDL-1)	*10F.9G2*	*149Sm*
TCRβ	*H57-597*	*150Nd*
Ly6C	*HK1.4*	*151Eu*
CD3	*145-2C11*	*152Sm*
CD172a/SIRPα	*P84*	*153Eu*
CD103	*2E7*	*154Sm*
CD106/VCAM-1	*429*	*155Gd*
CD19	*6D5*	*156Gd*
Ly6G	*1A8*	*158Gd*
CD11b	*M1/70*	*160Gd*
CD54/ICAM-1	*YN1/1.7.4*	*161Dy*
CD24	*M1/69*	*162Dy*
CD8a	53-6.7	*164Dy*
CD45	*30-F11*	*165Ho*
CD86/B7-2	*GL-1*	*166Er*
CD25/IL-2Rα	*3C7*	*167Er*
FCeR1	*MAR*1*	*169Tm*
CD80	*3/23*	*170Er*
CD206	*C068C2*	*172Yb*
$H\text{-}2L^d/H\text{-}2D^b$ *(MHC I)*	*28-14-8*	*173Yb*
I-A/I-E (MHC II)	*M5/114.15.2*	*174Yb*
F4/80	*BM8*	*175Lu*

Supplementary Table 1. CyTOF Antibody Panel. Table lists the antigen, clone and coupled-metal of the antibodies that were used in our CyTOF immunophenotyping experiments.
