# Supplementary material for: Acute microglia ablation induces neurodegeneration in the somatosensory system
Source: Nat Commun. 2018 Nov 1;9:4578. doi: 10.1038/s41467-018-05929-4 (PMC6212411; doi:10.1038/s41467-018-05929-4)
Supplement: Supplementary file 2 — Peer Review File [file 41467_2018_5929_MOESM2_ESM.pdf]

Editorial Note: This manuscript has been previously reviewed at another journal that is not operating a transparent peer review scheme. This document only contains reviewer comments and rebuttal letters for versions considered at Nature Communications.
